# Exploring inconsistencies in genome-wide protein function annotations: a machine learning approach

**DOI:** 10.1186/1471-2105-8-284

**Published:** 2007-08-03

**Authors:** Carson Andorf, Drena Dobbs, Vasant Honavar

**Affiliations:** 1 Artificial Intelligence Laboratory, Department of Computer Science, Iowa State University, Ames, Iowa, 50011, USA; 2 Department of Genetics, Development and Cell Biology, Iowa State University, Ames, Iowa, 50011, USA; 3 Bioinformatics and Computational Biology Graduate Program, Iowa State University, Ames, Iowa, 50011, USA; 4 Center for Computational Intelligence, Learning, and Discovery, Iowa State University, Ames, Iowa, 50011, USA

## Abstract

**Background:**

Incorrectly annotated sequence data are becoming more commonplace as databases increasingly rely on automated techniques for annotation. Hence, there is an urgent need for computational methods for checking consistency of such annotations against independent sources of evidence and detecting potential annotation errors. We show how a machine learning approach designed to automatically predict a protein's Gene Ontology (GO) functional class can be employed to identify potential gene annotation errors.

**Results:**

In a set of 211 previously annotated mouse protein kinases, we found that 201 of the GO annotations returned by AmiGO appear to be *inconsistent *with the UniProt functions assigned to their human counterparts. In contrast, 97% of the predicted annotations generated using a machine learning approach were *consistent *with the UniProt annotations of the human counterparts, as well as with available annotations for these mouse protein kinases in the Mouse Kinome database.

**Conclusion:**

We conjecture that most of our predicted annotations are, therefore, correct and suggest that the machine learning approach developed here could be routinely used to detect potential errors in GO annotations generated by high-throughput gene annotation projects.

Editors Note : Authors from the original publication (Okazaki et al.: *Nature *2002, **420**:563–73) have provided their response to Andorf et al, directly following the correspondence.

## Background

As more genomic sequences become available, functional annotation of genes presents one of the most important challenges in bioinformatics. Because experimental determination of protein structure and function is expensive and time-consuming, there is an increasing reliance on automated approaches to assignment of Gene Ontology (GO) [1] functional categories to protein sequences. An advantage of such automated methods is that they can be used to annotate hundreds or thousands of proteins in a matter of minutes, which makes their use especially attractive – if not unavoidable – in large-scale genome-wide annotation efforts.

Most automated approaches to protein function annotation rely on transfer of annotations from previously annotated proteins, based on sequence or structural similarity. Such annotations are susceptible to several sources of error, including errors in the original annotations from which new annotations are inferred, errors in the algorithms, bugs in the programs or scripts used to process the data, clerical errors on the part of human curators, among others. The effect of such errors can be magnified because they can propagate from one set of annotated sequences to another through widespread use of automated techniques for genome-wide functional annotation of proteins [2-5]. Once introduced, such errors can go undetected for a long time. Because of the increasing reliance of biologists and computational biologists on reliable functional annotations for formulation of hypotheses, design of experiments, and interpretation of results, incorrect annotations can lead to wasted effort and erroneous conclusions. Computational approaches to checking automatically inferred annotations against independent sources of evidence and detecting potential annotation errors offer a potential solution to this problem [6-11].

Previous work of several groups, including our own [12-19] has demonstrated the usefulness of machine learning approaches to assigning putative functions to proteins based on the amino acid sequence of the proteins. On the specific problem of predicting the catalytic activity of proteins from amino acid sequence, we showed that machine learning approaches outperform methods based on sequence homology [13]. This is especially true when sequence identity among proteins with a specified function is below 10%; the accuracy of predictions by our HDTree classifier was 8%–16% better than that of PSI-BLAST [13]. The discriminatory power of machine learning approaches thus suggests they should be valuable for detecting potential annotation errors in functional genomics databases.

Here we demonstrate that a machine learning approach, designed to predict GO functional classifications for proteins, can be used to identify and correct potential annotation errors. In this study, we focused on a small but clinically important subset of protein kinases, for which we "stumbled upon" potential annotation errors while evaluating the performance of protein function classification algorithms. We chose a set of protein kinases categorized under the GO class GO0004672, Protein Kinase Activity, which includes proteins with serine/threonine (Ser/Thr) kinase activity (GO0004674) and tyrosine (Tyr) kinase activity (GO0004713). Post-translational modification of proteins by phosphorylation plays an important regulatory role in virtually every signaling pathway in eukaryotic cells, modulating key biological processes associated with development and diseases including cancer, diabetes, hyperlipidemia and inflammation [20,21]. It is natural to expect that such well studied and functionally significant families of protein kinases are correctly annotated by genome-wide annotation efforts.

## Results

The initial aim of our experiments was to evaluate the effectiveness of machine learning approaches to automate sequence-based classification of protein kinases into subfamilies. Because both the Ser/Thr and Tyr subfamilies contain highly divergent members, some of which share less than 10% sequence identity with other members, they offer a rigorous test case for evaluating the potential general utility of this approach. Previously, we developed HDTree [13], a two-stage approach that combines a classifier based on amino acid *k*-gram composition of a protein sequence, with a classifier that relies on transfer of annotation from PSI-BLAST hits (see Methods for details). A protein kinase classifier was trained on a set of 330 human protein kinases from the Ser/Thr protein kinase (GO0004674) and Tyr protein kinase (GO0004713) functional classes based on direct and indirect annotations assigned by AmiGO [22], a valuable and widely used tool for retrieving GO functional annotations of proteins. Performance of the classifier was evaluated, using 10-fold cross-validation, on two datasets: i) the dataset of 330 *human *protein kinases, and ii) a dataset of 244 *mouse *protein kinases drawn from the same GO functional classes. The initial datasets were not filtered based on evidence codes or sequence identity cutoffs.

Using the AmiGO annotations as reference, the resulting HDTree classifier correctly distinguished between Ser/Thr kinases and Tyr kinases in the human kinase dataset with an overall accuracy of 89.1% and a kappa coefficient of 0.76. In striking contrast, the accuracy of the classifier on the mouse kinase dataset was only 15.1%; the correlation between the GO functional categories predicted by the classifier and the AmiGO reference labels was an alarming -0.40: 72 of the 244 mouse kinases were classified as Ser/Thr kinases, 105 as Tyr kinases, and 67 as "dual specificity" kinases (belonging to both GO0004674 and GO0004713 classes) (see Table 1).

**Table 1:** Performance of classifiers trained on human versus mouse kinases in predicting AmiGO annotations. The performance measures accuracy, kappa coefficient, correlation coefficient, precision, and recall are reported for two of the HDTree classifiers. The first classifier is trained on 330 human kinases. The performance is based on 10-fold cross-validation. The second classifier is trained on the 330 human kinases and tested on 244 mouse kinases. The annotations for the mouse and human kinases were obtained from AmiGO.

			**Correlation Coefficient**			**Precision**			**Recall**		
			
**Classifier**	**Accuracy**	**Kappa Coefficient**	**Ser/Thr**	**Tyr**	**Dual**	**Ser/Thr**	**Tyr**	**Dual**	**Ser/Thr**	**Tyr**	**Dual**
**Human**	89.1	0.76	0.82	0.86	0.30	0.97	1.00	0.15	0.95	0.74	0.71
**Mouse**	15.1	-0.40	-0.40	-0.43	-0.01	0.17	0.11	0.25	0.41	0.07	0.01

Assuming the AmiGO annotations were correct, these results suggested that either this particular machine learning approach is extremely ineffective for classifying mouse protein labels, or that human and mouse protein kinases have so little in common that a classifier trained on the human proteins is doomed to fail miserably on the mouse proteins. In light of the demonstrated effectiveness of machine learning approaches on a broad range of classification tasks that arise in bioinformatics [23], and well-documented high degree of homology between human and mouse proteins [24], neither of these conclusions seemed warranted. Could this discrepancy be explained by the AmiGO annotations for mouse protein kinases? We proceeded to investigate this possibility.

**Figure 1:**
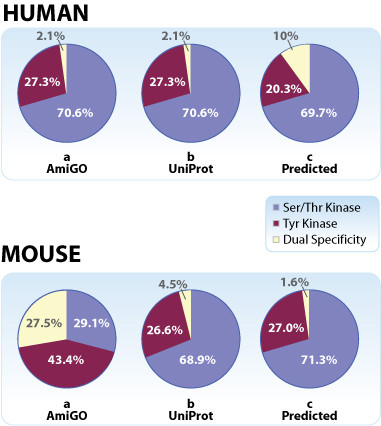
**Distribution of Ser/Thr, Tyr, and dual specificity kinases among annotated protein kinases in human versus mouse genomes **[see Additional file 9**]**. Pie charts illustrate the functional family distribution of protein kinases in human (top) versus mouse (bottom), based on: **a. AmiGO functional classifications**: Ser/Thr (GO0004674) [Blue]; Tyr (GO0004713) [Red] or "dual specificity" (proteins with both GO classifications) [Yellow]. **b. UniProt annotations**: classification based on UniProt records containing the key words Ser/Thr [Blue], Tyr [Red], or dual specificity [Yellow] [see Additional file 2]. **c. Predicted annotations by the HDTree classifier**: The classifier was built on human proteins with functional labels Ser/Thr (GO0004674) [Blue], Tyr (GO0004713) [Red] or "dual specificity" [Yellow] derived from AmiGO and verified by UniProt [see Additional file 4].

A comparison of the distribution of Ser/Thr, Tyr, and dual specificity kinases in mouse versus human (Figure 1a) reveals a striking discordance: based on AmiGO annotations, mouse has many more Tyr and dual specificity kinases than human and only 40% as many Ser/Thr protein kinases. In contrast, as explained below, the fractions of Ser/Thr, Tyr, and dual specificity kinases based on UniProt annotations are very similar in mouse and human (Figure 1b). Furthermore, the predictions of our two-stage machine learning algorithm are in good agreement with the UniProt annotations for both human and mouse protein kinases (Figures 1b and 1c, and Additional File 9).

**Figure 2:**
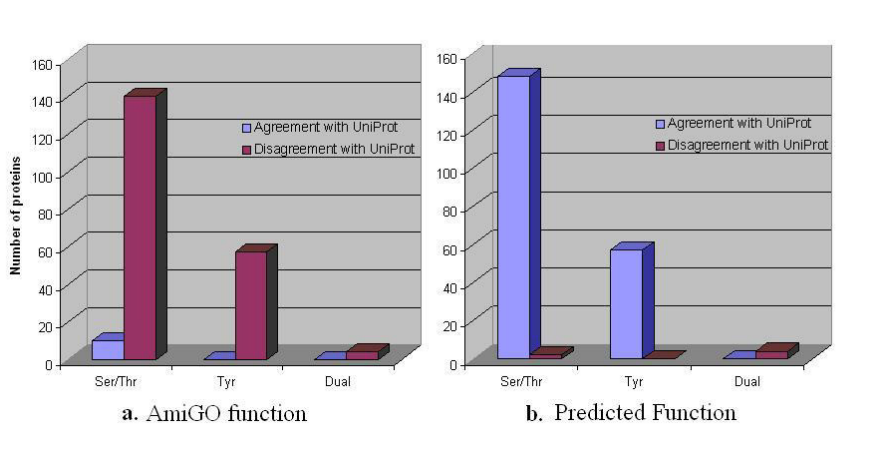
**Comparison of UniProt annotations of mouse protein kinase sequences with annotations from AmiGO or predicted by HDTree**. The bar charts illustrate the number of proteins that were in agreement (blue)/disagreement (red) with the annotations found in UniProt. Proteins that belong to each of the three functional classes found in the UniProt records are represented by two bars. The blue bar represents the number of proteins in which UniProt and the given method share the same annotation (*agreement*) for that function. The red bar represents the number of proteins in which UniProt and the given method have different annotations (*disagreement*) for that function. **a**. AmiGO vs. UniProt annotations **b**. HDTree predictions vs. UniProt annotations [see Additional files 3 and 4].

Examination of the GO evidence codes for the mouse protein kinases revealed that 211 of 244 mouse protein kinases included the evidence code "RCA," "inferred from reviewed computational analysis" [see Additional file 1], indicating that these annotations had been assigned using computational tools and reviewed by a human curator before being deposited in the database used by AmiGO. Notably, 28 of 33 (85%) mouse protein kinases with an evidence code other than RCA (e.g., "inferred from direct assay") were assigned "correct" labels, relative to the AmiGO reference, by the classifier trained on the human protein kinase data. Each of the 211 proteins with the RCA evidence code had at least one annotation that could be traced to the FANTOM Consortium and RIKEN Genome Exploration Research Group [25], a source of protein function annotations in the Mouse Genome Database (MGD) [24]. To further examine each of these 211 mouse protein kinases, we used the gene IDs obtained from AmiGO to extract information about each protein from UniProt [26]. We searched the UniProt records for mention of "Serine/Threonine" or "Tyrosine" (or their synonyms) in fields for protein name, synonyms, references, similarity, keywords, or function, and created a dataset in which each protein kinase had one of the corresponding UniProt labels: "Ser/Thr kinase," "Tyr kinase," or "dual specificity kinase" if both keywords were found. Results of our comparison of UniProt labels with AmiGO annotations for each class in this dataset of 211 mouse protein kinases are shown in Figure 2a: for 201 of the 211 cases with an RCA annotation code, the UniProt and AmiGO labels were inconsistent. Results of our comparison are shown in Table 2 [see Additional files 2 and 3].

**Table 2:** Comparison of AmiGO and UniProt annotations for 211 mouse protein kinases with RCA Evidence code. Each of the 211 mouse kinase proteins with an RCA evidence code used in this study has both an AmiGO and a UniProt annotation. This table shows the number of proteins that have each of the nine possible combinations of AmiGO and UniProt annotations. Each row of the table represents one of the three possible UniProt labels and each column represents each of the three AmiGO annotations. Each entry of the table shows the number of proteins with the corresponding annotation. Note that all entries along the diagonal (in bold) show the number of proteins for which the AmiGO and UniProt annotations were in agreement. All other entries show the number of proteins where AmiGO and UniProt were in disagreement [see Additional files 2 and 3].

**KINASE FAMILY**	**AmiGO Ser/Thr**	**AmiGO Tyr**	**AmiGO Dual specificity**
**UniProt Ser/Thr**	**10**	105	35
**UniProt Tyr**	54	**0**	3
**UniProt Dual specificity**	0	4	**0**

This result led us to test the ability of the HDTree classifier trained on the human kinase dataset to correctly predict the family classifications for proteins in the mouse kinase dataset, this time using UniProt instead of AmiGO annotations as the "correct" reference labels. Strikingly, the classifier (trained on the human kinase dataset) achieved a classification accuracy of 97.2%, with a kappa coefficient of 0.93, on the mouse kinase dataset. As illustrated in Figure 2b, the classifier correctly classified 205 out of the 211 mouse kinases into Ser/Thr, Tyr or dual specificity classes compared with 10 out of 211 for AmiGO. A direct comparison of classifiers based on UniProt annotations and AmiGO annotations can be seen in Table 3. This performance actually exceeded that of the same classifier tested on the human kinase dataset, for which an overall classification accuracy of 89.1%, with a kappa coefficient of 0.76, was obtained [see Table 1 and see Additional file 4]

**Table 3:** Comparison of performance of classifiers based on AmiGO annotations and UniProt annotations. The performance measures accuracy, kappa coefficient, correlation coefficient, precision, and recall are reported for two of the HDTree classifiers. Both classifiers were trained on 330 human kinases and tested on 211 mouse kinases with RCA evidence codes in AmiGO. The first classifier was trained and tested with annotations provided by UniProt and the second classifier used annotations obtained from AmiGO.

			**Correlation Coefficient**			**Precision**			**Recall**		
			
**Classifier**	**Accuracy**	**Kappa Coefficient**	**Ser/Thr**	**Tyr**	**Dual**	**Ser/Thr**	**Tyr**	**Dual**	**Ser/Thr**	**Tyr**	**Dual**
**UniProt**	97.1	0.93	0.98	0.94	0.00	0.97	0.97	0.00	0.99	1.00	0.00
**AmiGO**	4.2	-0.37	-0.64	-0.85	0.00	0.06	0.00	0.00	0.14	0.00	0.00

The HDTree method uses a decision tree built from the output from eight individual classifiers. A decision tree is built by selecting, in a greedy fashion, the individual classifier that provides the maximum information about the class label at each step, [27]. By examining the decision tree, it is easy to identify the individual classifiers that have the greatest influence on the classification. In the case of the kinase datasets used in this study, the classifiers constructed by the NB(k) algorithms using trimers and quadmers, NB(3) and NB(4), were found to provide the most information regarding class labels. This suggests that the biological "signals" detected by these classifiers are groups of 3–4 residues, not necessarily contiguous in the primary amino acid sequence, but often in close proximity or interacting within three-dimensional structures to form functional sites (e.g., catalytic sites, binding sites), an idea supported by the results of our previous work [13]. Notably, the NB(3) and NB(4) classifiers appear to contribute more to the ability to distinguish proteins with very closely related enzymatic activities than PSI-BLAST. The PSI-BLAST results influenced the final classification, however, when the NB(3) and NB(4) classifiers disagreed on the classification.

## Discussion

Examination of the Mouse Kinome Database [28] reveals that the majority of annotated mouse kinases have a human ortholog with sequence identity > 90% [see Additional files 5 and 6]. The results summarized in Figures 1 and 2, together with the assumption that the relative proportions of Ser/Thr, Tyr and dual specificity kinases should not be significant different in human and mouse, led us to conclude that UniProt derived annotations are more likely to be correct than those returned by AmiGO for this group of mouse protein kinases with the RCA evidence code. We have shared our findings with the Mouse Genome Database [24], which is in the process of identifying and rectifying the source of potential problems with these annotations.

Identifying potential annotation errors in a specific dataset such as the mouse kinase dataset solves only a part of a larger problem. Because annotation errors can propagate across multiple databases through the widespread – and often necessary – use of information derived from available annotations, it is important to track and correct errors in other databases that rely on the erroneous source. For example, using AmiGO, we retrieved 136 rat protein kinases for which annotations had been transferred from mouse protein kinases based on homology (indicated by the evidence code "ISS," 'inferred from sequence or structural similarity') with one of the 201 erroneously annotated mouse protein kinases. Examination of the UniProt records for these 136 rat protein kinases revealed that 94 of those labeled as "Ser/Thr" kinases by UniProt had AmiGO annotations of "Tyr" or "dual specificity" kinase, and 42 of those labeled as "Tyr" kinases by UniProt had AmiGO annotations of "Ser/Thr" or "dual specificity" kinase [see Additional files 7 and 8].

A recent study found that the GO annotations with ISS (inferred from sequence or structural similarity) evidence code could have error rates as high as 49% [29]. This argues for the development and large-scale application of a suite of computational tools for identifying and flagging potentially erroneous annotations in functional genomics databases. Our results suggest the utility of including machine learning methods among such a suite of tools. Large-scale application of machine learning tools to protein annotation has to overcome several challenges. Because many proteins are multi-functional, classifiers should be able to assign a sequence to multiple, not mutually exclusive, classes (the *multi label *classification problem), or more generally, to a subset of nodes in a directed-acyclic graph, e.g., the GO hierarchy, (the *structured label *classification problem). Fortunately, a number of research groups have developed machine learning algorithms for multi-label and structured label classification and demonstrated their application in large-scale protein function classification [30-33]. We can draw on recent advances in machine learning methods for hierarchical multi-label classification of large sequence datasets to adapt our method to work in such a setting. For example, a binary classifier can be trained to determine membership of a given sequence in the class represented by each node of the GO hierarchy, starting with the root node (to which trivially the entire dataset is assigned). Binary classifiers at each node in the hierarchy can then be trained recursively, focusing on the dataset passed to that node from its parent(s) in the GO hierarchy.

In this study, we have limited our attention to *sequence-based *machine learning methods for annotation of protein sequences. With the increasing availability of other types of data (protein structure, gene expression profiles, etc.), there is a growing interest in machine learning and other computational methods for genome-wide prediction of protein function using diverse types of information [34-39]. Such techniques can be applied in a manner similar to our use of sequence-based machine learning to identify potentially erroneous annotations in existing databases.

## Conclusion

The increasing reliance on automated tools in genome-wide functional annotation of proteins has led to a corresponding increase in the risk of propagation of annotation errors across genome databases. Short of direct experimental validation of every annotation, it is impossible to ensure that the annotations are accurate. The results presented here and in recent related studies [6-11] underscore the need for checking the consistency of annotations against multiple sources of information and carefully exploring the sources of any detected inconsistencies. Addressing this problem requires the use of machine readable metadata that capture precise descriptions of all data sources, data provenance, background assumptions, and algorithms used to infer the derived information. There is also a need for computational tools that can detect annotation inconsistencies and alert data sources and their users regarding potential errors. Expertly curated databases such as the Mouse Genome Database are indispensable for research in functional genomics and systems biology, and it is important to emphasize that several measures for finding and correcting inconsistent annotations are already in place at MGD [24]. The present study suggests that additional measures, especially in the case of protein annotations with RCA evidence code, can further increase the reliability of these valuable resources.

## Methods

### Classification Strategy

We constructed an HDTree binary classifier, described below, for each of the three kinase families. The first two kinase families correspond to the GO labels GO0004674 (Ser/Thr kinases) or GO0004713 (Tyr kinases) but not both; the third family corresponds to dual-specificity kinases that belong to both GO0004674 and GO0004713. Classifier #1 distinguishes between Ser/Thr kinases and the rest (Tyr and dual-specificity kinases). Similarly, classifier #2 distinguishes between Tyr kinases and the rest (Ser/Thr and dual specificity kinases). Classifier #3 distinguishes dual-specificity kinases from the rest (those with only Ser/Thr or Tyr activity), based on the predictions generated by classifier #1 and classifier #2 as follows: If only classifier #1 generates a positive prediction, the corresponding sequence is classified as (exclusively) a Ser/Thr kinase. If only classifier #2 generates a positive prediction, the corresponding sequence is classified as (exclusively) Tyr kinase. If both classifiers generate a positive prediction or if both classifiers generate a negative prediction, the corresponding sequence is classified as a dual-specificity kinase. We interpret the disagreement between the classifiers as indicative of signaling evidence that the protein is neither exclusively Ser/Thr nor Tyr, and hence, likely to have dual specificity. More sophisticated evidence combination methods could be used instead. However, this simple technique worked sufficiently well in the case of this dataset (see Table 4).

**Table 4:** Classification schema for Classier #3 (Method for predicting dual specificity kinases). HDTree Classifier #3 uses the outputs from HDTree Classifier #1 and HDTree Classifier #2 to distinguish between dual-specificity kinases, Ser/Thr kinases, and Tyr kinases. There are four possible labelings from the binary classifiers #1 and #2. 'Yes' or 'No' votes from Classifier #1 correspond to predictions of Ser/Thr or Tyr labels, respectively, for the protein. 'Yes' or 'No' votes from Classifier #2 correspond to predictions of Tyr or Ser/Thr labels. When both classifiers predict the protein to be Ser/Thr (that is, Classifier #1 votes 'Yes' and Classifier #2 votes 'No'), Classifier #3 labels the protein as "exclusively Ser/Thr" (and hence, not Tyr). Similarly, when both classifiers predict the protein to be Tyr, Classifier #3 labels the protein as "exclusively Tyr" (and hence not Ser/Thr). When both classifiers vote 'Yes' or when both vote 'No,' Classifier #3 labels the protein as having "Dual" catalytic activity. See Methods section for details on each classifier.

Prediction of classifier #1 (Ser/Thr)	Prediction of classifier #2 (Tyr)	**New Prediction of classifier #3 (Dual, Ser/Thr, Tyr)**
Yes	Yes	**Dual**
Yes	No	**exclusively Ser/Thr**
No	Yes	**exclusively Tyr**
No	No	**Dual**

### HDTree Method

As noted above, an HDTree binary classifier [13] is constructed for each of the three kinase families. Each HDTree binary classifier is a decision tree classifier that assigns a class label to a target sequence based on the binary class labels output by the Naïve Bayes, NB k-gram, NB(k), and PSI-BLAST classifiers for the corresponding kinase families. Because there are eight classifiers Naïve Bayes, NB 2-gram, NB 3-gram, NB 4-gram, NB(2), NB(3), NB(4), and PSI-BLAST, the input to a HDTree binary classifier for each kinase family consists of an 8-tuple of class labels assigned to the sequence by the corresponding 8 classifiers. The output of the HDTree classifier for kinase family *c *is a binary class label (1 if the predicted class is *c*; 0 otherwise). Thus, each HDTree classifier is a decision tree classifier that is trained to predict the binary class label of a query sequence based on the 8-tuple of class labels predicted by the eight individual classifiers. Because HDTree is a decision tree, it is easy to determine which individual classifier(s) provided the most information in regards to the predicted class label. In the resulting tree, nodes near the top of the tree provided the most information about the class label. Thus, HDTree can also facilitate identification of the determinative biological sequence signals. We used the Weka version 3.4.4 implementation [40] (J4.8) of the C4.5 decision tree learning algorithm [27].

We describe below, a class of probabilistic models for sequence classification.

### Classification Using a Probabilistic Model

We start by introducing the general procedure for building a classifier from a probabilistic generative model.

Suppose we can specify a probabilistic model *α *for sequences defined over some alphabet Σ (which in our case is the 20-letter amino acid alphabet). The model *α *specifies for any sequence S¯
 MathType@MTEF@5@5@+=feaafiart1ev1aaatCvAUfKttLearuWrP9MDH5MBPbIqV92AaeXatLxBI9gBaebbnrfifHhDYfgasaacH8akY=wiFfYdH8Gipec8Eeeu0xXdbba9frFj0=OqFfea0dXdd9vqai=hGuQ8kuc9pgc9s8qqaq=dirpe0xb9q8qiLsFr0=vr0=vr0dc8meaabaqaciaacaGaaeqabaqabeGadaaakeaadaqdaaqaaiabdofatbaaaaa@2DEC@ = *s*_1_, ..., *s*_*n*_, the probability *P*_*α*_(S¯
 MathType@MTEF@5@5@+=feaafiart1ev1aaatCvAUfKttLearuWrP9MDH5MBPbIqV92AaeXatLxBI9gBaebbnrfifHhDYfgasaacH8akY=wiFfYdH8Gipec8Eeeu0xXdbba9frFj0=OqFfea0dXdd9vqai=hGuQ8kuc9pgc9s8qqaq=dirpe0xb9q8qiLsFr0=vr0=vr0dc8meaabaqaciaacaGaaeqabaqabeGadaaakeaadaqdaaqaaiabdofatbaaaaa@2DEC@ = *s*_1_, ..., *s*_*n*_) of generating the sequence S¯
 MathType@MTEF@5@5@+=feaafiart1ev1aaatCvAUfKttLearuWrP9MDH5MBPbIqV92AaeXatLxBI9gBaebbnrfifHhDYfgasaacH8akY=wiFfYdH8Gipec8Eeeu0xXdbba9frFj0=OqFfea0dXdd9vqai=hGuQ8kuc9pgc9s8qqaq=dirpe0xb9q8qiLsFr0=vr0=vr0dc8meaabaqaciaacaGaaeqabaqabeGadaaakeaadaqdaaqaaiabdofatbaaaaa@2DEC@. Suppose we assume that sequences belonging to class *c*_*j *_are generated by the probabilistic generative model *α *(*c*_*j*_).

Then, Pα(S¯=s1,...,sn|cj)=Pα(cj)(S¯=s1,...,sn)
 MathType@MTEF@5@5@+=feaafiart1ev1aaatCvAUfKttLearuWrP9MDH5MBPbIqV92AaeXatLxBI9gBaebbnrfifHhDYfgasaacH8akY=wiFfYdH8Gipec8Eeeu0xXdbba9frFj0=OqFfea0dXdd9vqai=hGuQ8kuc9pgc9s8qqaq=dirpe0xb9q8qiLsFr0=vr0=vr0dc8meaabaqaciaacaGaaeqabaqabeGadaaakeaacqWGqbaudaWgaaWcbaacciGae8xSdegabeaakiabcIcaOmaanaaabaGaem4uamfaaiabg2da9iabdohaZnaaBaaaleaacqaIXaqmaeqaaOGaeiilaWIaeiOla4IaeiOla4IaeiOla4IaeiilaWIaem4Cam3aaSbaaSqaaiabd6gaUbqabaGccqGG8baFcqWGJbWydaWgaaWcbaGaemOAaOgabeaakiabcMcaPiabg2da9iabdcfaqnaaBaaaleaacqWFXoqycqGGOaakcqWGJbWydaWgaaadbaGaemOAaOgabeaaliabcMcaPaqabaGccqGGOaakdaqdaaqaaiabdofatbaacqGH9aqpcqWGZbWCdaWgaaWcbaGaeGymaedabeaakiabcYcaSiabc6caUiabc6caUiabc6caUiabcYcaSiabdohaZnaaBaaaleaacqWGUbGBaeqaaOGaeiykaKcaaa@58AE@ is the probability of S¯
 MathType@MTEF@5@5@+=feaafiart1ev1aaatCvAUfKttLearuWrP9MDH5MBPbIqV92AaeXatLxBI9gBaebbnrfifHhDYfgasaacH8akY=wiFfYdH8Gipec8Eeeu0xXdbba9frFj0=OqFfea0dXdd9vqai=hGuQ8kuc9pgc9s8qqaq=dirpe0xb9q8qiLsFr0=vr0=vr0dc8meaabaqaciaacaGaaeqabaqabeGadaaakeaadaqdaaqaaiabdofatbaaaaa@2DEC@ given that the class is *c*_*j*_. Therefore, given the probabilistic generative model for each of the classes in *C *(the set of possible mutually exclusive class labels) for sequences over the alphabet Σ, we can compute the most likely class label *c*(S¯
 MathType@MTEF@5@5@+=feaafiart1ev1aaatCvAUfKttLearuWrP9MDH5MBPbIqV92AaeXatLxBI9gBaebbnrfifHhDYfgasaacH8akY=wiFfYdH8Gipec8Eeeu0xXdbba9frFj0=OqFfea0dXdd9vqai=hGuQ8kuc9pgc9s8qqaq=dirpe0xb9q8qiLsFr0=vr0=vr0dc8meaabaqaciaacaGaaeqabaqabeGadaaakeaadaqdaaqaaiabdofatbaaaaa@2DEC@) for any given sequence S¯
 MathType@MTEF@5@5@+=feaafiart1ev1aaatCvAUfKttLearuWrP9MDH5MBPbIqV92AaeXatLxBI9gBaebbnrfifHhDYfgasaacH8akY=wiFfYdH8Gipec8Eeeu0xXdbba9frFj0=OqFfea0dXdd9vqai=hGuQ8kuc9pgc9s8qqaq=dirpe0xb9q8qiLsFr0=vr0=vr0dc8meaabaqaciaacaGaaeqabaqabeGadaaakeaadaqdaaqaaiabdofatbaaaaa@2DEC@ = *s*_1_, ..., *s*_*n *_as follows: c(S¯)=arg⁡max⁡cj∈CPα(S¯=s1,...,sn|cj)P(cj)
 MathType@MTEF@5@5@+=feaafiart1ev1aaatCvAUfKttLearuWrP9MDH5MBPbIqV92AaeXatLxBI9gBaebbnrfifHhDYfgasaacH8akY=wiFfYdH8Gipec8Eeeu0xXdbba9frFj0=OqFfea0dXdd9vqai=hGuQ8kuc9pgc9s8qqaq=dirpe0xb9q8qiLsFr0=vr0=vr0dc8meaabaqaciaacaGaaeqabaqabeGadaaakeaacqWGJbWycqGGOaakdaqdaaqaaiabdofatbaacqGGPaqkcqGH9aqpcyGGHbqycqGGYbGCcqGGNbWzdaWfqaqaaiGbc2gaTjabcggaHjabcIha4bWcbaGaem4yam2aaSbaaWqaaiabdQgaQbqabaWccqGHiiIZcqWGdbWqaeqaaOGaemiuaa1aaSbaaSqaaGGaciab=f7aHbqabaGccqGGOaakdaqdaaqaaiabdofatbaacqGH9aqpcqWGZbWCdaWgaaWcbaGaeGymaedabeaakiabcYcaSiabc6caUiabc6caUiabc6caUiabcYcaSiabdohaZnaaBaaaleaacqWGUbGBaeqaaOGaeiiFaWNaem4yam2aaSbaaSqaaiabdQgaQbqabaGccqGGPaqkcqWGqbaucqGGOaakcqWGJbWydaWgaaWcbaGaemOAaOgabeaakiabcMcaPaaa@5B08@. Hence, the goal of a machine learning algorithm for sequence classification is to estimate the parameters that describe the corresponding probabilistic models from data. Different classifiers differ with regard to their ability to capture the dependencies among the elements of a sequence.

In what follows, we use the following notations.

*n *= S¯
 MathType@MTEF@5@5@+=feaafiart1ev1aaatCvAUfKttLearuWrP9MDH5MBPbIqV92AaeXatLxBI9gBaebbnrfifHhDYfgasaacH8akY=wiFfYdH8Gipec8Eeeu0xXdbba9frFj0=OqFfea0dXdd9vqai=hGuQ8kuc9pgc9s8qqaq=dirpe0xb9q8qiLsFr0=vr0=vr0dc8meaabaqaciaacaGaaeqabaqabeGadaaakeaadaqdaaqaaiabdofatbaaaaa@2DEC@ = the length of the sequence |S¯
 MathType@MTEF@5@5@+=feaafiart1ev1aaatCvAUfKttLearuWrP9MDH5MBPbIqV92AaeXatLxBI9gBaebbnrfifHhDYfgasaacH8akY=wiFfYdH8Gipec8Eeeu0xXdbba9frFj0=OqFfea0dXdd9vqai=hGuQ8kuc9pgc9s8qqaq=dirpe0xb9q8qiLsFr0=vr0=vr0dc8meaabaqaciaacaGaaeqabaqabeGadaaakeaadaqdaaqaaiabdofatbaaaaa@2DEC@|

*k *= the size of the k-gram (k-mer) used in the model

*s*_*i *_= the *i*^*th*^element in the sequence S¯
 MathType@MTEF@5@5@+=feaafiart1ev1aaatCvAUfKttLearuWrP9MDH5MBPbIqV92AaeXatLxBI9gBaebbnrfifHhDYfgasaacH8akY=wiFfYdH8Gipec8Eeeu0xXdbba9frFj0=OqFfea0dXdd9vqai=hGuQ8kuc9pgc9s8qqaq=dirpe0xb9q8qiLsFr0=vr0=vr0dc8meaabaqaciaacaGaaeqabaqabeGadaaakeaadaqdaaqaaiabdofatbaaaaa@2DEC@

*c*_*j *_= the *j*^*th *^class in the class set *C*

### Naïve Bayes Classifier

The Naïve Bayes classifier assumes that each element of the sequence is independent of the other elements given the class label. Consequently,

c(S¯)=arg⁡max⁡cj∈CPα∏i=1nPα(s1|cj)⋅⋅⋅Pα(sn|cj)P(cj)
 MathType@MTEF@5@5@+=feaafiart1ev1aaatCvAUfKttLearuWrP9MDH5MBPbIqV92AaeXatLxBI9gBaebbnrfifHhDYfgasaacH8akY=wiFfYdH8Gipec8Eeeu0xXdbba9frFj0=OqFfea0dXdd9vqai=hGuQ8kuc9pgc9s8qqaq=dirpe0xb9q8qiLsFr0=vr0=vr0dc8meaabaqaciaacaGaaeqabaqabeGadaaakeaacqWGJbWycqGGOaakdaqdaaqaaiabdofatbaacqGGPaqkcqGH9aqpcyGGHbqycqGGYbGCcqGGNbWzdaWfqaqaaiGbc2gaTjabcggaHjabcIha4bWcbaGaem4yam2aaSbaaWqaaiabdQgaQbqabaWccqGHiiIZcqWGdbWqaeqaaOGaemiuaa1aaSbaaSqaaGGaciab=f7aHbqabaGcdaqeWbqaaiabdcfaqnaaBaaaleaacqWFXoqyaeqaaOGaeiikaGIaem4Cam3aaSbaaSqaaiabigdaXaqabaGccqGG8baFcqWGJbWydaWgaaWcbaGaemOAaOgabeaakiabcMcaPiabgwSixlabgwSixlabgwSixlabdcfaqnaaBaaaleaacqWFXoqyaeqaaOGaeiikaGIaem4Cam3aaSbaaSqaaiabd6gaUbqabaGccqGG8baFcqWGJbWydaWgaaWcbaGaemOAaOgabeaakiabcMcaPaWcbaGaemyAaKMaeyypa0JaeGymaedabaGaemOBa4ganiabg+GivdGccqWGqbaucqGGOaakcqWGJbWydaWgaaWcbaGaemOAaOgabeaakiabcMcaPaaa@6E2B@

Note that the Naive Bayes classifier for sequences treats each sequence as though it were simply a *bag *of letters. We now consider two Naive Bayes-like models based on *k*-grams.

### Naïve Bayes *k*-grams Classifier

The Naive Bayes *k*-grams (NB *k*-grams) [12,13,41] method uses a sliding a window of size *k *along each sequence to generate a *bag *of *k*-grams representation of the sequence. Much like in the case of the Naive Bayes classifier described above treats each *k*-gram in the bag to be independent of the others given the class label for the sequence. Given this probabilistic model, the standard method for classification using a probabilistic model can be applied. The probability model associated with Naïve Bayes *k*-grams:

Pα(S¯=[S1=s1,...,Sn=sn])=arg⁡max⁡cj∈CPα∏i=1n−k+1Pα(Si=si,...,Si+k−1=si+k−1|cj)P(cj)
 MathType@MTEF@5@5@+=feaafiart1ev1aaatCvAUfKttLearuWrP9MDH5MBPbIqV92AaeXatLxBI9gBaebbnrfifHhDYfgasaacH8akY=wiFfYdH8Gipec8Eeeu0xXdbba9frFj0=OqFfea0dXdd9vqai=hGuQ8kuc9pgc9s8qqaq=dirpe0xb9q8qiLsFr0=vr0=vr0dc8meaabaqaciaacaGaaeqabaqabeGadaaakeaacqWGqbaudaWgaaWcbaacciGae8xSdegabeaakiabcIcaOmaanaaabaGaem4uamfaaiabg2da9iabcUfaBjabdofatnaaBaaaleaacqaIXaqmaeqaaOGaeyypa0Jaem4Cam3aaSbaaSqaaiabigdaXaqabaGccqGGSaalcqGGUaGlcqGGUaGlcqGGUaGlcqGGSaalcqWGtbWudaWgaaWcbaGaemOBa4gabeaakiabg2da9iabdohaZnaaBaaaleaacqWGUbGBaeqaaOGaeiyxa0LaeiykaKIaeyypa0JagiyyaeMaeiOCaiNaei4zaC2aaCbeaeaacyGGTbqBcqGGHbqycqGG4baEaSqaaiabdogaJnaaBaaameaacqWGQbGAaeqaaSGaeyicI4Saem4qameabeaakiabdcfaqnaaBaaaleaacqWFXoqyaeqaaOWaaebCaeaacqWGqbaudaWgaaWcbaGae8xSdegabeaakiabcIcaOiabdofatnaaBaaaleaacqWGPbqAaeqaaOGaeyypa0Jaem4Cam3aaSbaaSqaaiabdMgaPbqabaGccqGGSaalcqGGUaGlcqGGUaGlcqGGUaGlcqGGSaalcqWGtbWudaWgaaWcbaGaemyAaKMaey4kaSIaem4AaSMaeyOeI0IaeGymaedabeaakiabg2da9iabdohaZnaaBaaaleaacqWGPbqAcqGHRaWkcqWGRbWAcqGHsislcqaIXaqmaeqaaOGaeiiFaWNaem4yam2aaSbaaSqaaiabdQgaQbqabaGccqGGPaqkaSqaaiabdMgaPjabg2da9iabigdaXaqaaiabd6gaUjabgkHiTiabdUgaRjabgUcaRiabigdaXaqdcqGHpis1aOGaemiuaaLaeiikaGIaem4yam2aaSbaaSqaaiabdQgaQbqabaGccqGGPaqkaaa@8D59@

A problem with the NB *k*-grams approach is that successive *k*-grams extracted from a sequence share *k*-1 elements in common. This grossly and systematically violates the independence assumption of Naive Bayes.

### Naïve Bayes (k)

We introduce the Naive Bayes (*k*) or the NB(*k*) model [12,13,41] to explicitly model the dependencies that arise as a consequence of the overlap between successive *k*-grams in a sequence. We represent the dependencies in a graphical form by drawing edges between the elements that are directly dependent on each other.

Using the Junction Tree Theorem for graphical models [42], it can be proved [41] that the correct probability model *α *that captures the dependencies among overlapping *k*-grams is given by:

Pα(S¯=[S1=s1,...,Sn=sn])=∏i=1n−k+1Pα(Si=si,...,Si+k−1=si+k−1)∏i=2n−k+1Pα(Si=si,...,Si+k−2=si+k−2)
MathType@MTEF@5@5@+=feaafiart1ev1aaatCvAUfKttLearuWrP9MDH5MBPbIqV92AaeXatLxBI9gBaebbnrfifHhDYfgasaacH8akY=wiFfYdH8Gipec8Eeeu0xXdbba9frFj0=OqFfea0dXdd9vqai=hGuQ8kuc9pgc9s8qqaq=dirpe0xb9q8qiLsFr0=vr0=vr0dc8meaabaqaciaacaGaaeqabaqabeGadaaakeaacqWGqbaudaWgaaWcbaacciGae8xSdegabeaakiabcIcaOmaanaaabaGaem4uamfaaiabg2da9iabcUfaBjabdofatnaaBaaaleaacqaIXaqmaeqaaOGaeyypa0Jaem4Cam3aaSbaaSqaaiabigdaXaqabaGccqGGSaalcqGGUaGlcqGGUaGlcqGGUaGlcqGGSaalcqWGtbWudaWgaaWcbaGaemOBa4gabeaakiabg2da9iabdohaZnaaBaaaleaacqWGUbGBaeqaaOGaeiyxa0LaeiykaKIaeyypa0ZaaSaaaeaadaqeWaqaaiabdcfaqnaaBaaaleaacqWFXoqyaeqaaOGaeiikaGIaem4uam1aaSbaaSqaaiabdMgaPbqabaGccqGH9aqpcqWGZbWCdaWgaaWcbaGaemyAaKgabeaakiabcYcaSiabc6caUiabc6caUiabc6caUiabcYcaSiabdofatnaaBaaaleaacqWGPbqAcqGHRaWkcqWGRbWAcqGHsislcqaIXaqmaeqaaOGaeyypa0Jaem4Cam3aaSbaaSqaaiabdMgaPjabgUcaRiabdUgaRjabgkHiTiabigdaXaqabaGccqGGPaqkaSqaaiabdMgaPjabg2da9iabigdaXaqaaiabd6gaUjabgkHiTiabdUgaRjabgUcaRiabigdaXaqdcqGHpis1aaGcbaWaaebmaeaacqWGqbaudaWgaaWcbaGae8xSdegabeaakiabcIcaOiabdofatnaaBaaaleaacqWGPbqAaeqaaOGaeyypa0Jaem4Cam3aaSbaaSqaaiabdMgaPbqabaGccqGGSaalcqGGUaGlcqGGUaGlcqGGUaGlcqGGSaalcqWGtbWudaWgaaWcbaGaemyAaKMaey4kaSIaem4AaSMaeyOeI0IaeGOmaidabeaakiabg2da9iabdohaZnaaBaaaleaacqWGPbqAcqGHRaWkcqWGRbWAcqGHsislcqaIYaGmaeqaaOGaeiykaKcaleaacqWGPbqAcqGH9aqpcqaIYaGmaeaacqWGUbGBcqGHsislcqWGRbWAcqGHRaWkcqaIXaqma0Gaey4dIunaaaaaaa@9BCD@

Now, given this probabilistic model, we can use the standard approach to classification given a probabilistic model. It is easily seen that when k = 1, Naive Bayes 1-grams as well as Naive Bayes (1) reduce to the Naive Bayes model.

The relevant probabilities required for specifying the above models can be estimated using standard techniques for estimation of probabilities using Laplace estimators [43].

### PSI-Blast

We used PSI-BLAST (from the latest release of BLAST) [44] to construct a binary classifier for each class. We used the binary class label predicted by the PSI-BLAST based classifier as an additional input to our HD-Tree classifier. Given a query sequence to be classified, we use PSI-BLAST to compare the query sequence against a reference protein sequence database, i.e., the training set used in the cross-validation process. We run PSI-BLAST with the query sequence against the reference database. We assign to the query sequence the functional class of the top scoring hit (the sequence with the lowest e-value) from the PSI-BLAST results. The resulting binary prediction of the PSI-BLAST classifier for class *c *is 1 if the class label for the top scoring hit is *c*. Otherwise, it is 0. An e-value cut-off of 0.0001 was used for PSI-BLAST, with all other parameters set to their default values.

### Performance Evaluation

The performance measures [45] used to evaluate each of the different classifiers trained using machine learning algorithms are summarized in Tables 5 and 6.

**Table 5:** Performance measure definitions for binary classification. The performance measures *accuracy, precision, recall, correlation coefficient, and kappa coefficient*are used to evaluate the performance of our machine learning approaches [45]. *Accuracy *is the fraction of overall predictions that are correct. *Precision *is the ratio of predicted true positive examples to the total number of actual positive examples. *Recall *is the ratio of predicted true positives to the total number of examples predicted as positive. *Correlation coefficient *measures the correlation between predictions and actual class labels. *Kappa coefficient *is used as a measure of agreement between two random variables (predictions and actual class labels). The table summarizes the definitions of performance measures in the 2-class setting (binary classification), where *M *= the total number of classes and *N *= the total number of examples. *TP, TN, FP, FN *are the true positives, true negatives, false positives, and false negatives for the given confusion matrix.

Performance Measure	Definition
Accuracy	TP+TNTP+FP+TN+FN MathType@MTEF@5@5@+=feaafiart1ev1aaatCvAUfKttLearuWrP9MDH5MBPbIqV92AaeXatLxBI9gBaebbnrfifHhDYfgasaacH8akY=wiFfYdH8Gipec8Eeeu0xXdbba9frFj0=OqFfea0dXdd9vqai=hGuQ8kuc9pgc9s8qqaq=dirpe0xb9q8qiLsFr0=vr0=vr0dc8meaabaqaciaacaGaaeqabaqabeGadaaakeaadaWcaaqaaiabdsfaujabdcfaqjabgUcaRiabdsfaujabd6eaobqaaiabdsfaujabdcfaqjabgUcaRiabdAeagjabdcfaqjabgUcaRiabdsfaujabd6eaojabgUcaRiabdAeagjabd6eaobaaaaa@3E1C@
Precision	TPTP+FN MathType@MTEF@5@5@+=feaafiart1ev1aaatCvAUfKttLearuWrP9MDH5MBPbIqV92AaeXatLxBI9gBaebbnrfifHhDYfgasaacH8akY=wiFfYdH8Gipec8Eeeu0xXdbba9frFj0=OqFfea0dXdd9vqai=hGuQ8kuc9pgc9s8qqaq=dirpe0xb9q8qiLsFr0=vr0=vr0dc8meaabaqaciaacaGaaeqabaqabeGadaaakeaadaWcaaqaaiabdsfaujabdcfaqbqaaiabdsfaujabdcfaqjabgUcaRiabdAeagjabd6eaobaaaaa@348C@
Recall	TPTP+FP MathType@MTEF@5@5@+=feaafiart1ev1aaatCvAUfeBSjuyZL2yd9gzLbvyNv2CaerbwvMCKfMBHbqedmvETj2BSbqee0evGueE0jxyaibaieIgFLIOYR2NHOxjYhrPYhrPYpI8F4rqqrFfpeea0xe9Lq=Jc9vqaqpepm0xbbG8FasPYRqj0=yi0lXdbba9pGe9qqFf0dXdHuk9fr=xfr=xfrpiWZqaaeaabiGaaiaacaqabeaabeqacmaaaOqaamaalaaabaGaamivaiaadcfaaeaacaWGubGaamiuaiabgUcaRiaadAeacaWGqbaaaaaa@3B6B@
Correlation Coefficient	TP*TN−FP*FN(TP+FN)(TP+FP)(TN+FP)(TN+FN) MathType@MTEF@5@5@+=feaafiart1ev1aaatCvAUfKttLearuWrP9MDH5MBPbIqV92AaeXatLxBI9gBaebbnrfifHhDYfgasaacH8akY=wiFfYdH8Gipec8Eeeu0xXdbba9frFj0=OqFfea0dXdd9vqai=hGuQ8kuc9pgc9s8qqaq=dirpe0xb9q8qiLsFr0=vr0=vr0dc8meaabaqaciaacaGaaeqabaqabeGadaaakeaadaWcaaqaaiabdsfaujabdcfaqjabcQcaQiabdsfaujabd6eaojabgkHiTiabdAeagjabdcfaqjabcQcaQiabdAeagjabd6eaobqaamaakaaabaGaeiikaGIaemivaqLaemiuaaLaey4kaSIaemOrayKaemOta4KaeiykaKIaeiikaGIaemivaqLaemiuaaLaey4kaSIaemOrayKaemiuaaLaeiykaKIaeiikaGIaemivaqLaemOta4Kaey4kaSIaemOrayKaemiuaaLaeiykaKIaeiikaGIaemivaqLaemOta4Kaey4kaSIaemOrayKaemOta4KaeiykaKcaleqaaaaaaaa@5544@
Kappa Coefficient	(TP*+TN)−((TP+FN)*(TP+FP)+(TN+FN)*(TN+FP))N−((TP+FN)*(TP+FP)+(TN+FN)*(TN+FP)) MathType@MTEF@5@5@+=feaafiart1ev1aaatCvAUfKttLearuWrP9MDH5MBPbIqV92AaeXatLxBI9gBaebbnrfifHhDYfgasaacH8akY=wiFfYdH8Gipec8Eeeu0xXdbba9frFj0=OqFfea0dXdd9vqai=hGuQ8kuc9pgc9s8qqaq=dirpe0xb9q8qiLsFr0=vr0=vr0dc8meaabaqaciaacaGaaeqabaqabeGadaaakeaadaWcaaqaaiabcIcaOiabdsfaujabdcfaqjabcQcaQiabgUcaRiabdsfaujabd6eaojabcMcaPiabgkHiTiabcIcaOiabcIcaOiabdsfaujabdcfaqjabgUcaRiabdAeagjabd6eaojabcMcaPiabcQcaQiabcIcaOiabdsfaujabdcfaqjabgUcaRiabdAeagjabdcfaqjabcMcaPiabgUcaRiabcIcaOiabdsfaujabd6eaojabgUcaRiabdAeagjabd6eaojabcMcaPiabcQcaQiabcIcaOiabdsfaujabd6eaojabgUcaRiabdAeagjabdcfaqjabcMcaPiabcMcaPaqaaiabd6eaojabgkHiTiabcIcaOiabcIcaOiabdsfaujabdcfaqjabgUcaRiabdAeagjabd6eaojabcMcaPiabcQcaQiabcIcaOiabdsfaujabdcfaqjabgUcaRiabdAeagjabdcfaqjabcMcaPiabgUcaRiabcIcaOiabdsfaujabd6eaojabgUcaRiabdAeagjabd6eaojabcMcaPiabcQcaQiabcIcaOiabdsfaujabd6eaojabgUcaRiabdAeagjabdcfaqjabcMcaPiabcMcaPaaaaaa@79B3@

**Table 6:** Performance measure definitions for multi-class classification. The performance measures *accuracy, precision, recall, correlation coefficient, and kappa coefficient *are used to evaluate the performance of our machine learning approaches [45]. *Accuracy *is the fraction of overall predictions that are correct. *Precision *is the ratio of predicted true positive examples to the total number of actual positive examples. *Recall *is the ratio of predicted true positives to the total number of examples predicted as positive. *Correlation coefficient *measures the correlation between predictions and actual class labels. *Kappa coefficient *is used as a measure of agreement between two random variables (predictions and actual class labels). The table displays the general definition of each measure, where *M *= the total number of classes and *N *= the total number of examples, *x*_*ik *_represents the number of examples in row *i *and column *k *of the given confusion matrix.

Performance Measure	Definition
Accuracy (class *i*)	∑i=1MxiiN MathType@MTEF@5@5@+=feaafiart1ev1aaatCvAUfKttLearuWrP9MDH5MBPbIqV92AaeXatLxBI9gBaebbnrfifHhDYfgasaacH8akY=wiFfYdH8Gipec8Eeeu0xXdbba9frFj0=OqFfea0dXdd9vqai=hGuQ8kuc9pgc9s8qqaq=dirpe0xb9q8qiLsFr0=vr0=vr0dc8meaabaqaciaacaGaaeqabaqabeGadaaakeaadaWcaaqaamaaqadabaGaemiEaG3aaSbaaSqaaiabdMgaPjabdMgaPbqabaaabaGaemyAaKMaeyypa0JaeGymaedabaGaemyta0eaniabggHiLdaakeaacqWGobGtaaaaaa@38B1@
Precision (class *i*)	xii∑k=1Mxki MathType@MTEF@5@5@+=feaafiart1ev1aaatCvAUfKttLearuWrP9MDH5MBPbIqV92AaeXatLxBI9gBaebbnrfifHhDYfgasaacH8akY=wiFfYdH8Gipec8Eeeu0xXdbba9frFj0=OqFfea0dXdd9vqai=hGuQ8kuc9pgc9s8qqaq=dirpe0xb9q8qiLsFr0=vr0=vr0dc8meaabaqaciaacaGaaeqabaqabeGadaaakeaadaWcaaqaaiabdIha4naaBaaaleaacqWGPbqAcqWGPbqAaeqaaaGcbaWaaabmaeaacqWG4baEdaWgaaWcbaGaem4AaSMaemyAaKgabeaaaeaacqWGRbWAcqGH9aqpcqaIXaqmaeaacqWGnbqta0GaeyyeIuoaaaaaaa@3BEF@
Recall (class *i*)	xii∑k=1Mxik MathType@MTEF@5@5@+=feaafiart1ev1aaatCvAUfKttLearuWrP9MDH5MBPbIqV92AaeXatLxBI9gBaebbnrfifHhDYfgasaacH8akY=wiFfYdH8Gipec8Eeeu0xXdbba9frFj0=OqFfea0dXdd9vqai=hGuQ8kuc9pgc9s8qqaq=dirpe0xb9q8qiLsFr0=vr0=vr0dc8meaabaqaciaacaGaaeqabaqabeGadaaakeaadaWcaaqaaiabdIha4naaBaaaleaacqWGPbqAcqWGPbqAaeqaaaGcbaWaaabmaeaacqWG4baEdaWgaaWcbaGaemyAaKMaem4AaSgabeaaaeaacqWGRbWAcqGH9aqpcqaIXaqmaeaacqWGnbqta0GaeyyeIuoaaaaaaa@3BEF@
Correlation Coefficient (class *i*)	(xii*∑h≠ixhh)−(∑k=1Mxki*∑j=1Mxij)(xii+∑k=1Mxki)(xii+∑j=1Mxij)(∑h≠ixhh+∑k=1Mxki)(∑h≠ixhh+∑j=1Mxij) MathType@MTEF@5@5@+=feaafiart1ev1aaatCvAUfKttLearuWrP9MDH5MBPbIqV92AaeXatLxBI9gBaebbnrfifHhDYfgasaacH8akY=wiFfYdH8Gipec8Eeeu0xXdbba9frFj0=OqFfea0dXdd9vqai=hGuQ8kuc9pgc9s8qqaq=dirpe0xb9q8qiLsFr0=vr0=vr0dc8meaabaqaciaacaGaaeqabaqabeGadaaakeaadaWcaaqaaiabcIcaOiabdIha4naaBaaaleaacqWGPbqAcqWGPbqAaeqaaOGaeiOkaOYaaabeaeaacqWG4baEdaWgaaWcbaGaemiAaGMaemiAaGgabeaaaeaacqWGObaAcqGHGjsUcqWGPbqAaeqaniabggHiLdGccqGGPaqkcqGHsislcqGGOaakdaaeWaqaaiabdIha4naaBaaaleaacqWGRbWAcqWGPbqAaeqaaOGaeiOkaOYaaabmaeaacqWG4baEdaWgaaWcbaGaemyAaKMaemOAaOgabeaakiabcMcaPaWcbaGaemOAaOMaeyypa0JaeGymaedabaGaemyta0eaniabggHiLdaaleaacqWGRbWAcqGH9aqpcqaIXaqmaeaacqWGnbqta0GaeyyeIuoaaOqaamaakaaabaGaeiikaGIaemiEaG3aaSbaaSqaaiabdMgaPjabdMgaPbqabaGccqGHRaWkdaaeWaqaaiabdIha4naaBaaaleaacqWGRbWAcqWGPbqAaeqaaaqaaiabdUgaRjabg2da9iabigdaXaqaaiabd2eanbqdcqGHris5aOGaeiykaKIaeiikaGIaemiEaG3aaSbaaSqaaiabdMgaPjabdMgaPbqabaGccqGHRaWkdaaeWaqaaiabdIha4naaBaaaleaacqWGPbqAcqWGQbGAaeqaaaqaaiabdQgaQjabg2da9iabigdaXaqaaiabd2eanbqdcqGHris5aOGaeiykaKIaeiikaGYaaabeaeaacqWG4baEdaWgaaWcbaGaemiAaGMaemiAaGgabeaaaeaacqWGObaAcqGHGjsUcqWGPbqAaeqaniabggHiLdGccqGHRaWkdaaeWaqaaiabdIha4naaBaaaleaacqWGRbWAcqWGPbqAaeqaaaqaaiabdUgaRjabg2da9iabigdaXaqaaiabd2eanbqdcqGHris5aOGaeiykaKIaeiikaGYaaabeaeaacqWG4baEdaWgaaWcbaGaemiAaGMaemiAaGgabeaaaeaacqWGObaAcqGHGjsUcqWGPbqAaeqaniabggHiLdGccqGHRaWkdaaeWaqaaiabdIha4naaBaaaleaacqWGPbqAcqWGQbGAaeqaaaqaaiabdQgaQjabg2da9iabigdaXaqaaiabd2eanbqdcqGHris5aOGaeiykaKcaleqaaaaaaaa@AB9F@
Kappa Coefficient	∑i=1Mxii−∑h=1M(∑k=1Mxkh*∑j=1Mxhj)N−∑h=1M(∑k=1Mxkh*∑j=1Mxhj) MathType@MTEF@5@5@+=feaafiart1ev1aaatCvAUfKttLearuWrP9MDH5MBPbIqV92AaeXatLxBI9gBaebbnrfifHhDYfgasaacH8akY=wiFfYdH8Gipec8Eeeu0xXdbba9frFj0=OqFfea0dXdd9vqai=hGuQ8kuc9pgc9s8qqaq=dirpe0xb9q8qiLsFr0=vr0=vr0dc8meaabaqaciaacaGaaeqabaqabeGadaaakeaadaWcaaqaamaaqadabaGaemiEaG3aaSbaaSqaaiabdMgaPjabdMgaPbqabaGccqGHsisldaaeWaqaaiabcIcaOmaaqadabaGaemiEaG3aaSbaaSqaaiabdUgaRjabdIgaObqabaGccqGGQaGkdaaeWaqaaiabdIha4naaBaaaleaacqWGObaAcqWGQbGAaeqaaOGaeiykaKcaleaacqWGQbGAcqGH9aqpcqaIXaqmaeaacqWGnbqta0GaeyyeIuoaaSqaaiabdUgaRjabg2da9iabigdaXaqaaiabd2eanbqdcqGHris5aaWcbaGaemiAaGMaeyypa0JaeGymaedabaGaemyta0eaniabggHiLdaaleaacqWGPbqAcqGH9aqpcqaIXaqmaeaacqWGnbqta0GaeyyeIuoaaOqaaiabd6eaojabgkHiTmaaqadabaGaeiikaGYaaabmaeaacqWG4baEdaWgaaWcbaGaem4AaSMaemiAaGgabeaakiabcQcaQmaaqadabaGaemiEaG3aaSbaaSqaaiabdIgaOjabdQgaQbqabaGccqGGPaqkaSqaaiabdQgaQjabg2da9iabigdaXaqaaiabd2eanbqdcqGHris5aaWcbaGaem4AaSMaeyypa0JaeGymaedabaGaemyta0eaniabggHiLdaaleaacqWGObaAcqGH9aqpcqaIXaqmaeaacqWGnbqta0GaeyyeIuoaaaaaaa@7820@

## Authors' contributions

CA conceived of and designed the study, carried out the data analysis and visualization, developed the Java computer code, and drafted the manuscript. DD and VH contributed to the design of the study, analysis and interpretation of results, and writing of the manuscript. All authors read and approved the final manuscript.

## Response from original authors

Masaaki Furuno^1,4^, David Hill^2,5^, Judith Blake^2,5^, Richard Baldarelli^2^, Piero Carninci^3,4^, and Yoshihide Hayashizaki ^1,3,4^

Addresses (^1^Functional RNA Research Program, RIKEN Frontier Research System, RIKEN Wako Institute, Wako, Japan. ^2^Mouse Genome Informatics Consortium, The Jackson Laboratory, Bar Harbor, Maine, United States of America. ^3^Genome Science Laboratory, Discovery Research Institute, RIKEN Wako Institute, Wako, Japan. ^4^Laboratory for Genome Exploration Research Group, RIKEN Genomic Sciences Center, Yokohama Institute, Kanagawa, Japan. ^5^Gene Ontology Consortium, The Jackson Laboratory, Bar Harbor, Maine, United States of America)

In this paper, the authors checked for potential Gene Ontology (GO) annotation errors using a machine learning approach. The authors' method identified a set of errors in GO annotations that relate to a very small subset of results from the 2001/2002 FANTOM2 analysis. These have subsequently been corrected.

We agree with the authors point about the importance of detecting the annotation errors. However, we believe that the errors the authors describe are exaggerated in importance as a result of the selection of datasets that they used and for the small set of genes that they studied. We will explain why they obtained these results, and we have identified a data curation change that has been implemented. However, we note such updates and revisions are a daily part of the work of large bio-informatics resources and of the work of the genome informatics community.

The strategy employed in FANTOM2 was appropriate and reflected the best strategy for mining large-scale functional information available at the time. In the computational analysis published in 2002 by the FANTOM2 Consortium, protein sequences were compared to other protein sequences and GO annotations were inferred from identical or highly similar proteins. GO annotations were also inferred from InterPro domains that were found in the coding regions of the proteins. The advanced analysis resulted in GO predictions for many proteins we knew nothing about at that time. A subset of the results of this landmark analysis were integrated into Mouse Genome Informatics after the FANTOM2 publication. This data set is important because it was the first analysis of this scale and complexity performed in mouse.

By retrieving annotations from AmiGO, Andorf *et al *restricted themselves to the subset of aggressively predicted FANTOM2 GO annotations while not considering high-quality FANTOM2 GO annotations that are represented in MGI using other automated methods. This is because AmiGO by policy does not display annotations inferred from automated methods. Much of the FANTOM data does not appear in AmiGO because it entered the regular MGI annotation stream and receives regular refreshing. As a result, this analysis casts a small subset of the FANTOM2 GO annotations in an unfair light. To obtain a fair analysis of all GO terms annotated at the time of FANTOM2, the original FANTOM2 data are available [46].

The results reported by Andorf *et al *remind us that conclusions based on a particular data set must be viewed in the context of a thorough understanding of how the data was generated and what is being represented in the set that is used for the analysis. The errors in GO annotation found by the authors are not due to general poor quality of FANTOM2 annotation. Rather, unique annotations from FANTOM2, as data associated with a publication, were not being comprehensively updated. We are reminded that any annotations based on computational methods must be regularly re-evaluated. MGI curators have now screened and updated the annotations for genes associated with protein tyrosine and protein serine/threonine kinase activities.

## Supplementary Material

Additional file 1 **Supplementary Table 1**: Evidence Codes for AmiGO annotations. A table displaying the Evidence Codes for AmiGO annotations of the mouse protein kinases used in this study. Click here for file

Additional file 2 **Supplementary Table 2**: AmiGO annotations versus UniProt annotations (with UniProt Evidence). A table comparing the annotations found in the AmiGO server with the annotations found in UniProt. Click here for file

Additional file 3 **Supplementary Table 3**: AmiGO labels, UniProt labels, and Predicted Labels for each mouse kinase protein. A table comparing the predicted annotations from our three machine learning classifiers with the annotations of AmiGO and UniProt. Click here for file

Additional file 4 **Supplementary Data**: Machine learning approaches to predict Gene Ontology and/or UniProt Functional labels. The data provided represent the results and performance of all the machine learning approaches used in this study. Click here for file

Additional file 5 **Supplementary Table 4**: Mouse kinases having a human ortholog. A table displaying the human orthologs for the mouse kinases used in this study. The table also displays the identity between these orthologs. Click here for file

Additional file 6 **Supplementary Table 5**: Number of mouse kinases having a specified level of sequence identity with their human orthologs. A table displaying the summary statistics of Supplementary Table 4. Click here for file

Additional file 7 **Supplementary Note**. Because there is only a non-curated reference to the work done on "Rat ISS GO annotations from MGI's mouse gene data," we provide the abstract and a link to the original reference report in this file. Click here for file

Additional file 8 **Supplementary Table 6**: The UniProt and AmiGO annotations for the rat kinase proteins with mouse orthologs. This table displays the UniProt and AmiGO annotations for rat kinase proteins that were annotated based on a mouse ortholog. Click here for file

Additional file 9 **Supplementary Table 7**: Distribution of protein classes for human and mouse proteins annotated by AmiGO, UniProt, and HDTree. This table is a representation of the data used in Figure 1 which is a pie chart showing the distribution of human and mouse protein classes based on annotations found in AmiGO, UniProt, and predicted by HDTree. Click here for file
