# Supplementary material for: Exploring inconsistencies in genome-wide protein function annotations: a machine learning approach
Source: BMC Bioinformatics. 2007 Aug 3;8:284. doi: 10.1186/1471-2105-8-284 (PMC1994202; doi:10.1186/1471-2105-8-284)
Supplement: Additional file 1 — Supplementary Table 1: Evidence Codes for AmiGO annotations. A table displaying the Evidence Codes for AmiGO annotations of the mouse protein kinases used in this study. [file 1471-2105-8-284-S1.pdf]

Supplementary Table 1:

Evidence Codes for AmiGO annotations of mouse protein kinases (See Table legend below).

Gene ID	AmiGO label	4674 Evidence Code	4713 Evidence Code
2610018G03Rik	4713		RCA
Acvr1b	4713		RCA
Acvr2a	4713		RCA
Acvr2b	4713		RCA
Acvrl1	4713		RCA
Adrbk1	4713		RCA
Akt1	4713		RCA
Alk	4674	RCA	
Araf	4713		RCA
Atm	4674	TAS	
Aurka	4713		RCA
Aurkb	4713		RCA
Axl	4674	RCA	
Blk	4674	RCA	
Bmpr1a	4713		RCA
Bmpr1b	4713		RCA
Bmpr2	4713		RCA
Bmx	4674	RCA	
Btk	4674 / 4713	RCA	IDA
Camk1	4713		RCA
Camk1d	4674	ISS	
Camk1g	4674	RCA	
Camk2a	4674	IMP	
Camk2b	4674 / 4713	IMP	RCA
Camk2g	4674 / 4713	IMP/RCA	RCA
Camk4	4674	TAS	
Camkk1	4674 / 4713	ISS	RCA
Ccrk	4674 / 4713	RCA	RCA
Cdc2a	4713		RCA
Cdc2l5	4674 / 4713	RCA	RCA
Cdk5	4674	IDA	
Cdk7	4674 / 4713	ISS	RCA
Cdk9	4713		RCA
Cdkl1	4674 / 4713	RCA	RCA
Cdkl3	4674 / 4713	RCA	RCA
Cdkl4	4674 / 4713	RCA	RCA
Chek1	4713		RCA
Chek2	4713		RCA
Chuk	4713		RCA
Cit	4674	IDA	
Clk1	4674 / 4713	IDA	IDA
Clk2	4713		IDA
Clk3	4713		IDA
Clk4	4713		IDA

Cpne3	4674	RCA	
Csf1r	4674	RCA	IEA
Csk	4674	RCA	
Csnk1d	4713		RCA
Csnk1e	4713		RCA
Csnk1g2	4713		RCA
Csnk2a2	4674 / 4713	RCA	RCA
Dapk2	4713		RCA
Dapk3	4713		RCA
Dcamkl2	4674 / 4713	RCA	RCA
Ddr1	4674	RCA	
Dmpk	4713		RCA
Dyrk1a	4713		ISS
Egfr	4713		IDA
Eif2ak1	4713		RCA
Eif2ak3	4713		RCA
Eif2ak4	4674 / 4713	IDA	RCA
Epha1	4674 / 4713	RCA	RCA
Epha2	4674	RCA	
Epha3	4674 / 4713	RCA	IDA
Epha4	4674	RCA	
Epha5	4674	RCA	
Epha6	4674	RCA	
Epha7	4674	RCA	
Epha8	4674	RCA	
Ephb2	4674 / 4713	RCA	IDA
Ephb3	4674 / 4713	RCA	TAS
Ephb4	4674	RCA	
Ephb6	4674	RCA	
Erbb2	4674 / 4713	RCA	RCA
Ern2	4713		RCA
Fgfr1	4674 / 4713	RCA	TAS
Fgfr2	4674	RCA	
Fgfr3	4713		IDA
Fgfr4	4674	RCA	
Fgr	4674	RCA	
Flt1	4674	RCA	
Flt3	4674	RCA	
Flt4	4674	RCA	
Fyn	4713		IDA
Gprk2l	4674 / 4713	RCA	RCA
Gprk5	4674 / 4713	RCA	RCA
Gprk6	4713		RCA
Grk1	4713		RCA
Gsg2	4674	IDA	
Gsk3b	4674	IDA	
Hck	4674	RCA	
Hipk2	4674	ISS	
Hipk3	4713		RCA
Hunk	4713		RCA

Ick	4674	IDA	
Igf1r	4674	RCA	
Ikbkb	4713		RCA
Ikbke	4674 / 4713	IDA	RCA
Ilk	4674	ISS	
Insrr	4674	RCA	
Irak3	4674 / 4713	RCA	RCA
Itk	4674	RCA	
Jak1	4713		IDA
Jak2	4674 / 4713	RCA	IDA
Jak3	4713		IDA
Kdr	4674	RCA	
Kit	4674 / 4713	RCA	IDA
Ksr1	4713		RCA
Lats1	4713		RCA
Lck	4674	RCA	
Limk1	4713		RCA
Lrrk1	4674 / 4713	RCA	RCA
Ltk	4674	RCA	
Lyn	4713		IDA
Map2k3	4713		RCA
Map2k5	4713		RCA
Map3k12	4713		RCA
Map3k14	4713		RCA
Map3k3	4713		RCA
Map3k4	4713		RCA
Map3k7	4713		RCA
Map3k8	4713		RCA
Map4k1	4674 / 4713	RCA	RCA
Map4k2	4713		RCA
Mapk1	4674 / 4713	ISS	RCA
Mapk10	4713		RCA
Mapk11	4713		RCA
Mapk12	4713		RCA
Mapk13	4713		RCA
Mapk14	4713		RCA
Mapk3	4713		RCA
Mapk7	4713		RCA
Mapk8	4713		RCA
Mapk9	4713		RCA
Mapkapk2	4713		RCA
Mapkapk5	4713		RCA
Mark1	4674 / 4713	RCA	RCA
Mark2	4713		RCA
Mast1	4713		RCA
Mast2	4674 / 4713	IDA	RCA
Mastl	4674	RCA	
Matk	4674	RCA	
Melk	4674 / 4713	ISS	RCA
Mertk	4674	RCA	

Met	4674 / 4713	RCA	IDA
Mknk1	4713		RCA
Mos	4713		RCA
Musk	4713		TAS
Mylk2	4674	RCA	
Nek11	4674 / 4713	RCA	RCA
Nek2	4713		RCA
Nek4	4713		RCA
Nek6	4674 / 4713	RCA	RCA
Nek7	4674	RCA	
Nlk	4674 / 4713	RCA	RCA
Npr1	4674 / 4713	RCA	RCA
Oxsr1	4674 / 4713	RCA	RCA
Pak1	4674 / 4713	ISS	RCA
Pak2	4674	ISS	
Pak3	4713		RCA
Pak4	4674 / 4713	RCA	RCA
Pak7	4674 / 4713	IDA / RCA	RCA
Pask	4674 / 4713	RCA	RCA
Pbk	4674 / 4713	ISS / RCA	RCA
Pctk1	4713		RCA
Pctk3	4713		RCA
Pdgfra	4674	RCA	
Pdgfrb	4674	RCA	
Pdpk1	4674	IDA	
Pftk1	4674 / 4713	ISS	RCA
Phkg1	4713		RCA
Pim1	4713		RCA
Pim2	4674 / 4713	IDA	RCA
Pink1	4713		RCA
Pkmyt1	4713		RCA
Pkn2	4674 / 4713	RCA	RCA
Plk1	4713		RCA
Plk2	4713		RCA
Plk4	4713		RCA
Pnck	4713		RCA
Prkaca	4674 / 4713	IDA	RCA
Prkca	4674 / 4713	IDA	RCA
Prkcb1	4674 / 4713	RCA	RCA
Prkcc	4674 / 4713	RCA	RCA
Prkch	4713		RCA
Prkci	4713		RCA
Prkcm	4713		RCA
Prkcz	4713		RCA
Prkg2	4713		RCA
Prkx	4674 / 4713	ISS	RCA
Prpf4b	4713		RCA
Ptk2	4674	RCA	
Ptk6	4674	IDA / RCA	
Pxk	4674	RCA	

Ret	4674 / 4713	RCA	TAS
Ripk1	4713		RCA
Ripk5	4674 / 4713	RCA	RCA
Rock1	4713		RCA
Ror1	4674 / 4713	RCA	TAS
Ror2	4674 / 4713	RCA	TAS
Rps6ka1	4713		RCA
Rps6ka3	4674	IDA	
Rps6ka5	4674 / 4713	RCA	RCA
Rps6kb2	4713		RCA
Rps6kl1	4674	RCA	
Sbk1	4674 / 4713	RCA / ISS	RCA
Sgk2	4713		RCA
Sgk3	4674	IDA	
Slk	4713		RCA
Snf1lk2	4674	IDA	
Snrk	4674 / 4713	RCA	RCA
Src	4674 / 4713	RCA	IMP
Srpk1	4713		RCA
Srpk2	4713		RCA
Stk10	4674 / 4713	TAS	RCA
Stk16	4713		RCA
Stk17b	4674 / 4713	RCA	RCA
Stk23	4674	IDA	
Stk32b	4713		RCA
Stk36	4674 / 4713	RCA	RCA
Stk38l	4674	ISS / RCA	
Syk	4674 / 4713	RCA	IDA
Tbk1	4674 / 4713	RCA	RCA
Tec	4674	RCA	
Tek	4674	RCA	
Tgfbr1	4713		RCA
Tgfbr2	4713		RCA
Tie1	4674	RCA	
Tlk1	4674 / 4713	RCA	RCA
Tlk2	4713		RCA
Tnk1	4674	RCA	
Tnk2	4674 / 4713	RCA	RCA
Tssk1	4713		RCA
Tssk2	4713		RCA
Tssk6	4674 / 4713	RCA	RCA
Ttbk2	4674	IDA	
Txk	4674	RCA	
Tyk2	4674	RCA	
Tyro3	4674	RCA	
Vrk1	4674	IDA	
Vrk2	4674	IDA	
Vrk3	4674	RCA	
Yes1	4674	RCA	
Zap70	4713		IDA

Legend for Supplementary Table 1:

Evidence Codes for AmiGO annotations

The evidence codes for all 244 mouse protein kinases used in this study are displayed in this table. All ***Mouse Gene ID*** numbers were obtained from the AmiGO protein record. The ***Mouse AmiGO Label*** field is "4713" (Tyr) if a query in AmiGO for the GO label GO0004713 returns the corresponding protein for mouse proteins, "4674" (Ser/Thr) if a query in AmiGO for the GO label GO0004674 returns the corresponding protein for mouse proteins, or "4674 / 4713" if a query in AmiGO for both GO labels GO0004674 and GO0004713 returns the corresponding protein. The ***4674 Evidence Code*** and ***4713 Evidence Code*** fields contain the evidence code(s)* provided by AmiGO for a given protein belonging to either the Gene Ontology family GO0004674 or GO0004713. If this field is empty, then the given protein was not included in the list of proteins returned by AmiGO for that Gene Ontology family.

*Examples of evidence codes provided by AmiGO:

- IC: Inferred by Curator
- IDA: Inferred from Direct Assay
- IEA: Inferred from Electronic Annotation
- IEP: Inferred from Expression Pattern
- IGI: Inferred from Genetic Interaction
- IMP: Inferred from Mutant Phenotype
- IPI: Inferred from Physical Interaction
- ISS: Inferred from Sequence or Structural Similarity
- NAS: Non-traceable Author Statement
- ND: No biological Data available
- RCA: inferred from Reviewed Computational Analysis
- TAS: Traceable Author Statement
- NR: Not Recorded

More details on evidence code: http://www.geneontology.org/GO.evidence.shtml
