# Supplementary material for: Exploring inconsistencies in genome-wide protein function annotations: a machine learning approach
Source: BMC Bioinformatics. 2007 Aug 3;8:284. doi: 10.1186/1471-2105-8-284 (PMC1994202; doi:10.1186/1471-2105-8-284)
Supplement: Additional file 2 — Supplementary Table 2: AmiGO annotations versus UniProt annotations (with UniProt Evidence). A table comparing the annotations found in the AmiGO server with the annotations found in UniProt. [file 1471-2105-8-284-S2.pdf]

Supplementary Table 2:

AmiGO annotations versus UniProt annotations [with UniProt Evidence] (See Table legend below)

Gene ID	AmiGO label	UniProt label	UniProt Evidence
2610018G03Rik	4713	4674	Serine/threonine-protein kinase MST4
Acvr1b	4713	4674	Serine/threonine-protein kinase
Acvr2a	4713	4674	Serine/threonine-protein kinase
Acvr2b	4713	4674	Serine/threonine-protein kinase
Acvrl1	4713	4674	Serine/threonine-protein kinase
Adrbk1	4713	4674	Serine/threonine-protein kinase
Akt1	4713	4674	RAC-alpha serine/threonine-protein kinase
Alk	4674	4713	ALK tyrosine kinase receptor precursor
Araf	4713	4674	A-Raf proto-oncogene serine/threonine-protein kinase
Atm	4674	4674	Serine/threonine-protein kinase
Aurka	4713	4674	Serine/threonine-protein kinase 6
Aurkb	4713	4674	Serine/threonine-protein kinase
Axl	4674	4713	Proto-oncogene tyrosine-protein kinase MER precursor
Blk	4674	4713	Tyrosine-protein kinase BLK
Bmpr1a	4713	4674	Serine/threonine-protein kinase
Bmpr1b	4713	4674	Serine/threonine-protein kinase
Bmpr2	4713	4674	Belongs to the Ser/Thr protein kinase family
Bmx	4674	4713	Cytoplasmic tyrosine-protein kinase BMX
Btk	4674 / 4713	4713	Tyrosine-protein kinase BTK
Camk1	4713	4674	Serine/threonine-protein kinase
Camk1d	4674	4674	Belongs to the Ser/Thr protein kinase family
Camk1g	4674	4674	Serine/threonine-protein kinase
Camk2a	4674	4674	Serine/threonine-protein kinase
Camk2b	4674 / 4713	4674	Serine/threonine-protein kinase
Camk2g	4674 / 4713	4674	Serine/threonine-protein kinase
Camk4	4674	4674	Belongs to the Ser/Thr protein kinase family
Camkk1	4674 / 4713	4674	Serine/threonine-protein kinase
Ccrk	4674 / 4713	4674	Serine/threonine-protein kinase
Cdc2a	4713	4674	Serine/threonine-protein kinase
Cdc2l5	4674 / 4713	4674	Serine/threonine-protein kinase
Cdk5	4674	4674	Belongs to the Ser/Thr protein kinase family
Cdk7	4674 / 4713	4674 / 4713	Protein-tyrosine kinase; Belongs to the Ser/Thr protein kinase
Cdk9	4713	4674 / 4713	Protein-tyrosine kinase; Belongs to the Ser/Thr protein kinase
Cdkl1	4674 / 4713	4674	Serine/threonine-protein kinase
Cdkl3	4674 / 4713	4674	Serine/threonine-protein kinase
Cdkl4	4674 / 4713	4674	Serine/threonine-protein kinase
Chek1	4713	4674	Serine/threonine-protein kinase
Chek2	4713	4674	Serine/threonine-protein kinase
Chuk	4713	4674	Serine/threonine-protein kinase
Cit	4674	4674 / 4713	Dual specificity protein kinase activity
Clk1	4674 / 4713	4674 / 4713	Phosphorylates serines, threonines and tyrosines
Clk2	4713	4674 / 4713	Tyrosine-protein kinase, Serine/threonine-protein kinase
Clk3	4713	4674 / 4713	Tyrosine-protein kinase, Serine/threonine-protein kinase
Clk4	4713	4674 / 4713	Tyrosine-protein kinase, Serine/threonine-protein kinase
Cpne3	4674	4674	Serine/threonine-protein phosphatase

Csf1r	4674	4713	protein tyrosine-kinase transmembrane receptor
Csk	4674	4713	Tyrosine-protein kinase CSK
Csnk1d	4713	4674	Serine/threonine-protein kinase
Csnk1e	4713	4674	Serine/threonine-protein kinase
Csnk1g2	4713	4674	Serine/threonine-protein kinase
Csnk2a2	4674 / 4713	4674	Serine/threonine-protein kinase
Dapk2	4713	4674	Belongs to the Ser/Thr protein kinase family
Dapk3	4713	4674	Belongs to the Ser/Thr protein kinase family
Dcamkl2	4674 / 4713	4674	Serine/threonine-protein kinase
Ddr1	4674	4713	Tyrosine kinase DDR
Dmpk	4713	4674	Belongs to the Ser/Thr protein kinase family
Dyrk1a	4713	4674 / 4713	Serine/threonine-protein kinase; Tyrosine-protein kinase
Egfr	4713	4713	Tyrosine-protein kinase
Eif2ak1	4713	4674	Serine/threonine-protein kinase
Eif2ak3	4713	4674	Serine/threonine-protein kinase
Eif2ak4	4674 / 4713	4674	Serine/threonine-protein kinase
Epha1	4674 / 4713	4713	Tyrosine-protein kinase receptor
Epha2	4674	4713	Tyrosine-protein kinase receptor
Epha3	4674 / 4713	4713	Tyrosine-protein kinase receptor
Epha4	4674	4713	Tyrosine-protein kinase receptor
Epha5	4674	4713	Tyrosine-protein kinase receptor
Epha6	4674	4713	Tyrosine-protein kinase receptor
Epha7	4674	4713	Tyrosine-protein kinase receptor
Epha8	4674	4713	Tyrosine-protein kinase receptor
Ephb2	4674 / 4713	4713	Tyrosine-protein kinase receptor
Ephb3	4674 / 4713	4713	Tyrosine-protein kinase receptor
Ephb4	4674	4713	Tyrosine-protein kinase receptor
Ephb6	4674	4713	Tyrosine-protein kinase receptor
Erbb2	4674 / 4713	4713	Receptor tyrosine-protein kinase erbB-2 precursor
Ern2	4713	4674	Serine/threonine-protein
Fgfr1	4674 / 4713	4713	Belongs to the Tyr protein kinase family
Fgfr2	4674	4713	Belongs to the Tyr protein kinase family
Fgfr3	4713	4713	Belongs to the Tyr protein kinase family
Fgfr4	4674	4713	Belongs to the Tyr protein kinase family
Fgr	4674	4713	Proto-oncogene tyrosine-protein kinase FGR
Flt1	4674	4713	Belongs to the Tyr protein kinase family
Flt3	4674	4713	Belongs to the Tyr protein kinase family
Flt4	4674	4713	Belongs to the Tyr protein kinase family
Fyn	4713	4713	Proto-oncogene tyrosine-protein kinase Fyn
Gprk2l	4674 / 4713	4674	Serine/threonine-protein kinase
Gprk5	4674 / 4713	4674	Serine/threonine-protein kinase
Gprk6	4713	4674	Serine/threonine-protein kinase
Grk1	4713	4674	Serine/threonine-protein kinase
Gsg2	4674	4674	Serine/threonine-protein kinase
Gsk3b	4674	4674	Belongs to the Ser/Thr protein kinase family
Hck	4674	4713	Tyrosine-protein kinase HCK
Hipk2	4674	4674	serine/threonine-protein kinase
Hipk3	4713	4674	Belongs to the Ser/Thr protein kinase family
Hunk	4713	4674	Serine/threonine-protein kinase MAK-V
Ick	4674	4674	Serine/threonine-protein kinase ICK

Igf1r	4674	4713	Belongs to the Tyr protein kinase family
Ikbkb	4713	4674	Serine/threonine-protein kinase
Ikbke	4674 / 4713	4674	Serine/threonine-protein kinase
Ilk	4674	4674	Serine/threonine-protein kinase
Insrr	4674	4713	Tyrosine-protein kinase
Irak3	4674 / 4713	4674	Serine/threonine-protein kinase
Itk	4674	4713	Tyrosine-protein kinase ITK/TSK
Jak1	4713	4713	Tyrosine-protein kinase
Jak2	4674 / 4713	4713	Tyrosine-protein kinase JAK2
Jak3	4713	4713	Tyrosine-protein kinase
Kdr	4674	4713	Has a tyrosine-protein kinase activity
Kit	4674 / 4713	4713	Tyrosine-protein kinase
Ksr1	4713	4674 / 4713	Ser_thr_pkin;Tyr_pkinase
Lats1	4713	4674	Serine/threonine-protein kinase LATS1
Lck	4674	4713	Proto-oncogene tyrosine-protein kinase LCK
Limk1	4713	4674	Serine/threonine-protein kinase
Lrrk1	4674 / 4713	4674	Leucine-rich repeat serine/threonine-protein kinase 1
Ltk	4674	4713	Leukocyte tyrosine kinase receptor precursor
Lyn	4713	4713	Tyrosine-protein kinase Lyn
Map2k3	4713	4674	serine/threonine-protein kinase
Map2k5	4713	4674	serine/threonine-protein kinase
Map3k12	4713	4674	serine/threonine-protein kinase
Map3k14	4713	4674	serine/threonine-protein kinase
Map3k3	4713	4674	serine/threonine-protein kinase
Map3k4	4713	4674	serine/threonine-protein kinase
Map3k7	4713	4674	serine/threonine-protein kinase
Map3k8	4713	4674	serine/threonine-protein kinase
Map4k1	4674 / 4713	4674	serine/threonine-protein kinase
Map4k2	4713	4674	serine/threonine-protein kinase
Mapk1	4674 / 4713	4674	serine/threonine-protein kinase
Mapk10	4713	4674	serine/threonine-protein kinase
Mapk11	4713	4674	serine/threonine-protein kinase
Mapk12	4713	4674	serine/threonine-protein kinase
Mapk13	4713	4674	serine/threonine-protein kinase
Mapk14	4713	4674	serine/threonine-protein kinase
Mapk3	4713	4674	serine/threonine-protein kinase
Mapk7	4713	4674	serine/threonine-protein kinase
Mapk8	4713	4674	serine/threonine-protein kinase
Mapk9	4713	4674	Serine/threonine-protein kinase
Mapkapk2	4713	4674	Belongs to the Ser/Thr protein kinase family
Mapkapk5	4713	4674	Belongs to the Ser/Thr protein kinase family
Mark1	4674 / 4713	4674	Serine/threonine-protein kinase
Mark2	4713	4674	Serine/threonine-protein kinase MARK2
Mast1	4713	4674	Microtubule-associated serine/threonine-protein kinase 1
Mast2	4674 / 4713	4674	serine/threonine-protein kinase
Mastl	4674	4674	serine/threonine-protein kinase
Matk	4674	4713	Megakaryocyte-associated tyrosine-protein kinase
Melk	4674 / 4713	4674	Serine/threonine-protein kinase
Mertk	4674	4713	Proto-oncogene tyrosine-protein kinase MER precursor
Met	4674 / 4713	4713	Tyrosine-protein kinase

Mknk1	4713	4674	MAP kinase-interacting serine/threonine-protein kinase 2
Mos	4713	4674	Proto-oncogene serine/threonine-protein kinase mos
Musk	4713	4713	Muscle, skeletal receptor tyrosine protein kinase precursor
Mylk2	4674	4674	Serine/threonine-protein kinase
Nek11	4674 / 4713	4674	Serine/threonine-protein kinase
Nek2	4713	4674	Serine/threonine-protein kinase Nek2
Nek4	4713	4674	Serine/threonine-protein kinase Nek3
Nek6	4674 / 4713	4674	Serine/threonine-protein kinase
Nek7	4674	4674	Serine/threonine-protein kinase
Nlk	4674 / 4713	4674	Serine/threonine kinase NLK
Npr1	4674 / 4713	4674	Serine/threonine-protein kinase
Oxsr1	4674 / 4713	4674	Serine/threonine-protein kinase
Pak1	4674 / 4713	4674	Serine/threonine-protein kinase
Pak2	4674	4674	Serine/threonine-protein kinase
Pak3	4713	4674	Serine/threonine-protein kinase PAK 3
Pak4	4674 / 4713	4674	Serine/threonine-protein kinase
Pak7	4674 / 4713	4674	Serine/threonine-protein kinase
Pask	4674 / 4713	4674	serine/threonine-protein kinase
Pbk	4674 / 4713	4674	Serine/threonine-protein kinase
Pctk1	4713	4674	Serine/threonine-protein kinase PCTAIRE-1
Pctk3	4713	4674	Serine/threonine-protein kinase PCTAIRE-3
Pdgfra	4674	4713	Tyrosine-protein kinase
Pdgfrb	4674	4713	Tyrosine-protein kinase
Pdpk1	4674	4674 / 4713	Phosphorylated on tyrosine and serine/threonine
Pftk1	4674 / 4713	4674	Serine/threonine-protein kinase
Phkg1	4713	4674	Serine/threonine-protein kinase
Pim1	4713	4674	Proto-oncogene serine/threonine-protein kinase Pim-1
Pim2	4674 / 4713	4674	Serine/threonine-protein kinase Pim-2
Pink1	4713	4674	Serine/threonine-protein kinase PINK1, mitochondrial precursor
Pkmyt1	4713	4674 / 4713	Membrane-associated tyrosine- and threonine-specific cdc2-inhibitory kinase
Pkn2	4674 / 4713	4674	Serine/threonine-protein kinase N2
Plk1	4713	4674	Serine/threonine-protein kinase PLK1
Plk2	4713	4674	Serine/threonine-protein kinase PLK2
Plk4	4713	4674	Serine/threonine-protein kinase PLK4
Pnck	4713	4674	Belongs to the Ser/Thr protein kinase family
Prkaca	4674 / 4713	4674	Belongs to the Ser/Thr protein kinase family
Prkca	4674 / 4713	4674	Serine/threonine-protein kinase
Prkcb1	4674 / 4713	4674	Serine/threonine-protein kinase
Prkcc	4674 / 4713	4674	Serine/threonine-protein kinase
Prkch	4713	4674	Serine/threonine-protein kinase
Prkci	4713	4674	Belongs to the Ser/Thr protein kinase family
Prkcm	4713	4674	Serine/threonine-protein kinase D1
Prkcz	4713	4674	Serine/threonine-protein kinase
Prkg2	4713	4674	Belongs to the Ser/Thr protein kinase family
Prkx	4674 / 4713	4674	Serine/threonine-protein kinase
Prpf4b	4713	4674	Serine/threonine-protein kinase
Ptk2	4674	4674	Serine/threonine-protein kinase PTK2/STK2
Ptk6	4674	4713	Tyrosine-protein kinase 6
Pxk	4674	4674	Ser_thr_pkinase

Ret	4674 / 4713	4713	Proto-oncogene tyrosine-protein kinase
Ripk1	4713	4674	Receptor-interacting serine/threonine-protein kinase 3
Ripk5	4674 / 4713	4674	Receptor-interacting serine/threonine-protein kinase 5
Rock1	4713	4674	Serine/threonine-protein kinase
Ror1	4674 / 4713	4713	Tyrosine-protein kinase transmembrane receptor
Ror2	4674 / 4713	4713	Tyrosine-protein kinase transmembrane receptor
Rps6ka1	4713	4674	Serine/threonine-protein kinase
Rps6ka3	4674	4674	Serine/threonine kinase
Rps6ka5	4674 / 4713	4674	Serine/threonine kinase
Rps6kb2	4713	4674	Serine/threonine-protein kinase
Rps6kl1	4674	4674	Belongs to the Ser/Thr protein kinase family
Sbk1	4674 / 4713	4674	Serine/threonine-protein kinase SBK1
Sgk2	4713	4674	Serine/threonine-protein kinase Sgk2
Sgk3	4674	4674	Serine/threonine-protein kinase Sgk3
Slk	4713	4674	STE20-like serine/threonine-protein kinase
Snf1lk2	4674	4674	Serine/threonine-protein kinase SNF1-like kinase 2
Snrk	4674 / 4713	4674	SNF-related serine/threonine-protein kinase
Src	4674 / 4713	4713	tyrosine-protein kinase
Srpk1	4713	4674	Serine/threonine-protein kinase
Srpk2	4713	4674	Serine/threonine-protein kinase
Stk10	4674 / 4713	4674	Serine/threonine-protein
Stk16	4713	4674	Serine/threonine-protein kinase 16
Stk17b	4674 / 4713	4674	Serine/threonine-protein
Stk23	4674	4674	Serine/threonine-protein
Stk32b	4713	4674	Serine/threonine-protein kinase 32B
Stk36	4674 / 4713	4674	Serine/threonine-protein kinase 36
Stk38l	4674	4674	Serine/threonine-protein
Syk	4674 / 4713	4713	Tyrosine-protein kinase SYK
Tbk1	4674 / 4713	4674	Serine/threonine-protein kinase TBK1
Tec	4674	4713	Tyrosine-protein kinase Tec
Tek	4674	4713	TEK receptor tyrosine kinase genes
Tgfbr1	4713	4674	Serine/threonine-protein kinase
Tgfbr2	4713	4674	Serine/threonine-protein kinase
Tie1	4674	4713	Tyrosine-protein kinase receptor Tie-1 precursor
Tlk1	4674 / 4713	4674	Serine/threonine-protein kinase tousled-like 1
Tlk2	4713	4674	Serine/threonine-protein kinase tousled-like 2
Tnk1	4674	4713	Non-receptor tyrosine-protein kinase TNK1
Tnk2	4674 / 4713	4713	Tyrosine kinase non-receptor protein 2
Tssk1	4713	4674	Testis-specific serine/threonine-protein kinase
Tssk2	4713	4674	Testis-specific serine/threonine-protein kinase
Tssk6	4674 / 4713	4674	Testis-specific serine/threonine-protein kinase 6
Ttbk2	4674	4674	Serine/threonine kinase
Txk	4674	4713	Tyrosine-protein kinase TXK
Tyk2	4674	4713	Non-receptor tyrosine-protein kinase TYK2
Tyro3	4674	4713	Tyrosine-protein kinase receptor TYRO3 precursor
Vrk1	4674	4674	Serine/threonine-protein kinase
Vrk2	4674	4674	Serine/threonine-protein kinase
Vrk3	4674	4674	Serine/threonine-protein kinase
Yes1	4674	4713	Proto-oncogene tyrosine-protein kinase Yes
Zap70	4713	4713	Tyrosine-protein kinase ZAP-70

Legend for Supplementary Table 2:

AmiGO annotations versus UniProt annotations (with UniProt Evidence)

This table displays the AmiGO annotation and UniProt annotations for each of the 244 mouse protein kinases used in this study. The ***Mouse Gene ID*** numbers were obtained from each of the AmiGO protein records. The ***AmiGO Label*** field is “4713” (Tyr) if a query in AmiGO for the GO label GO0004713 returns the corresponding protein for mouse proteins, “4674” (Ser/Thr) if a query in AmiGO for the GO label GO0004674 returns the corresponding protein for mouse proteins, or “4674 / 4713” if a query in AmiGO for both GO labels GO0004674 and GO0004713 returns the corresponding protein. The ***UniProt Label*** field is “ 4713” if a search in UniProt with the AmiGO Gene ID returns a mouse protein that contains a reference to the functional class protein-tyrosine kinase activity, ”4674” if a search in UniProt with the AmiGO Gene ID returns a mouse protein that contains a reference to the functional class serine/threonine kinase activity, or “4674 / 4713” if a search in UniProt returns a mouse protein that contains a reference to the functional class serine/threonine kinase activity and protein-tyrosine kinase activity or any evidence that would suggest dual specificity. The ***UniProt Evidence*** field contains at least one example of the evidence found in the UniProt record **(**within *protein name, synonyms, references, similarity, keywords*, or *function)* to support the label found in the *UniProt Label* field.
