# Supplementary material for: Exploring inconsistencies in genome-wide protein function annotations: a machine learning approach
Source: BMC Bioinformatics. 2007 Aug 3;8:284. doi: 10.1186/1471-2105-8-284 (PMC1994202; doi:10.1186/1471-2105-8-284)
Supplement: Additional file 3 — Supplementary Table 3: AmiGO labels, UniProt labels, and Predicted Labels for each mouse kinase protein. A table comparing the predicted annotations from our three machine learning classifiers with the annotations of AmiGO and UniProt. [file 1471-2105-8-284-S3.pdf]

Supplementary Table 3:

Comparison of AmiGO labels, UniProt labels, and Predicted Labels for each mouse kinase protein (See Table legend below)

Gene ID	AmiGO label	UniProt label	Prediction of Classifier #1	Prediction of Classifier #2	Prediction of Classifier #3
2610018G03Rik	4713	4674	4674	4674	4674
Acvr1b	4713	4674	4674	4674	4674
Acvr2a	4713	4674	4674	4674	4674
Acvr2b	4713	4674	4674	4674	4674
Acvrl1	4713	4674	4674	4674	4674
Adrbk1	4713	4674	4674	4674	4674
Akt1	4713	4674	4674	4674	4674
Alk	4674	4713	4713	4713	4713
Araf	4713	4674	4674	4674	4674
Atm	4674	4674	4674	4674	4674
Aurka	4713	4674	4674	4674	4674
Aurkb	4713	4674	4674	4674	4674
Axl	4674	4713	4713	4713	4713
Blk	4674	4713	4713	4713	4713
Bmpr1a	4713	4674	4674	4674	4674
Bmpr1b	4713	4674	4674	4674	4674
Bmpr2	4713	4674	4674	4674	4674
Bmx	4674	4713	4713	4713	4713
Btk	4674 / 4713	4713	4713	4713	4713
Camk1	4713	4674	4674	4674	4674
Camk1d	4674	4674	4674	4674	4674
Camk1g	4674	4674	4674	4674	4674
Camk2a	4674	4674	4674	4674	4674
Camk2b	4674 / 4713	4674	4674	4674	4674
Camk2g	4674 / 4713	4674	4674	4674	4674
Camk4	4674	4674	4674	4674	4674
Camkk1	4674 / 4713	4674	4674	4674	4674
Ccrk	4674 / 4713	4674	4674	4674	4674
Cdc2a	4713	4674	4674	4674	4674
Cdc2l5	4674 / 4713	4674	4674	4674	4674
Cdk5	4674	4674	4674	4674	4674
Cdk7	4674 / 4713	4674 / 4713	4674	4674	4674
Cdk9	4713	4674 / 4713	4674	4674	4674
Cdkl1	4674 / 4713	4674	4674	4674	4674
Cdkl3	4674 / 4713	4674	4674	4674	4674
Cdkl4	4674 / 4713	4674	4674	4674	4674
Chek1	4713	4674	4674	4674	4674
Chek2	4713	4674	4674	4674	4674
Chuk	4713	4674	4674	4674	4674
Cit	4674	4674 / 4713	4674	4674	4674
Clk1	4674 / 4713	4674 / 4713	4674	4713	4674/4713
Clk2	4713	4674 / 4713	4674	4674	4674
Clk3	4713	4674 / 4713	4674	4674	4674
Clk4	4713	4674 / 4713	4674	4713	4674/4713

Cpne3	4674	4674	4674	4674	4674
Csf1r	4674	4713	4713	4713	4713
Csk	4674	4713	4713	4713	4713
Csnk1d	4713	4674	4674	4674	4674
Csnk1e	4713	4674	4674	4674	4674
Csnk1g2	4713	4674	4674	4674	4674
Csnk2a2	4674 / 4713	4674	4674	4674	4674
Dapk2	4713	4674	4674	4674	4674
Dapk3	4713	4674	4674	4674	4674
Dcamkl2	4674 / 4713	4674	4674	4674	4674
Ddr1	4674	4713	4713	4713	4713
Dmpk	4713	4674	4674	4674	4674
Dyrk1a	4713	4674 / 4713	4674	4713	4674/4713
Egfr	4713	4713	4674	4713	4674/4713
Eif2ak1	4713	4674	4674	4674	4674
Eif2ak3	4713	4674	4674	4674	4674
Eif2ak4	4674 / 4713	4674	4674	4674	4674
Epha1	4674 / 4713	4713	4713	4713	4713
Epha2	4674	4713	4713	4713	4713
Epha3	4674 / 4713	4713	4713	4713	4713
Epha4	4674	4713	4713	4713	4713
Epha5	4674	4713	4713	4713	4713
Epha6	4674	4713	4713	4713	4713
Epha7	4674	4713	4713	4713	4713
Epha8	4674	4713	4713	4713	4713
Ephb2	4674 / 4713	4713	4713	4713	4713
Ephb3	4674 / 4713	4713	4713	4713	4713
Ephb4	4674	4713	4713	4713	4713
Ephb6	4674	4713	4713	4713	4713
Erbb2	4674 / 4713	4713	4713	4713	4713
Ern2	4713	4674	4674	4674	4674
Fgfr1	4674 / 4713	4713	4713	4713	4713
Fgfr2	4674	4713	4713	4713	4713
Fgfr3	4713	4713	4713	4713	4713
Fgfr4	4674	4713	4713	4713	4713
Fgr	4674	4713	4713	4713	4713
Flt1	4674	4713	4713	4713	4713
Flt3	4674	4713	4713	4713	4713
Flt4	4674	4713	4713	4713	4713
Fyn	4713	4713	4713	4713	4713
Gprk2l	4674 / 4713	4674	4674	4674	4674
Gprk5	4674 / 4713	4674	4674	4674	4674
Gprk6	4713	4674	4674	4674	4674
Grk1	4713	4674	4674	4674	4674
Gsg2	4674	4674	4674	4674	4674
Gsk3b	4674	4674	4674	4674	4674
Hck	4674	4713	4713	4713	4713
Hipk2	4674	4674	4674	4674	4674
Hipk3	4713	4674	4674	4674	4674
Hunk	4713	4674	4674	4674	4674

Ick	4674	4674	4674	4674	4674
Igf1r	4674	4713	4713	4713	4713
Ikbkb	4713	4674	4674	4674	4674
Ikbke	4674 / 4713	4674	4674	4674	4674
Ilk	4674	4674	4674	4674	4674
Insrr	4674	4713	4713	4713	4713
Irak3	4674 / 4713	4674	4674	4674	4674
Itk	4674	4713	4713	4713	4713
Jak1	4713	4713	4713	4713	4713
Jak2	4674 / 4713	4713	4713	4713	4713
Jak3	4713	4713	4713	4713	4713
Kdr	4674	4713	4713	4713	4713
Kit	4674 / 4713	4713	4713	4713	4713
Ksr1	4713	4674 / 4713	4674	4674	4674
Lats1	4713	4674	4674	4674	4674
Lck	4674	4713	4713	4713	4713
Limk1	4713	4674	4674	4674	4674
Lrrk1	4674 / 4713	4674	4674	4674	4674
Ltk	4674	4713	4713	4713	4713
Lyn	4713	4713	4713	4713	4713
Map2k3	4713	4674	4674	4674	4674
Map2k5	4713	4674	4674	4674	4674
Map3k12	4713	4674	4674	4674	4674
Map3k14	4713	4674	4674	4674	4674
Map3k3	4713	4674	4674	4674	4674
Map3k4	4713	4674	4674	4674	4674
Map3k7	4713	4674	4674	4674	4674
Map3k8	4713	4674	4674	4674	4674
Map4k1	4674 / 4713	4674	4674	4674	4674
Map4k2	4713	4674	4674	4674	4674
Mapk1	4674 / 4713	4674	4674	4674	4674
Mapk10	4713	4674	4674	4674	4674
Mapk11	4713	4674	4674	4674	4674
Mapk12	4713	4674	4674	4674	4674
Mapk13	4713	4674	4674	4674	4674
Mapk14	4713	4674	4674	4674	4674
Mapk3	4713	4674	4674	4674	4674
Mapk7	4713	4674	4674	4674	4674
Mapk8	4713	4674	4674	4674	4674
Mapk9	4713	4674	4674	4674	4674
Mapkapk2	4713	4674	4674	4674	4674
Mapkapk5	4713	4674	4674	4674	4674
Mark1	4674 / 4713	4674	4674	4674	4674
Mark2	4713	4674	4674	4674	4674
Mast1	4713	4674	4674	4674	4674
Mast2	4674 / 4713	4674	4674	4674	4674
Mastl	4674	4674	4674	4674	4674
Matk	4674	4713	4713	4713	4713
Melk	4674 / 4713	4674	4674	4674	4674
Mertk	4674	4713	4713	4713	4713

Met	4674 / 4713	4713	4713	4713	4713
Mknk1	4713	4674	4674	4674	4674
Mos	4713	4674	4674	4674	4674
Musk	4713	4713	4713	4713	4713
Mylk2	4674	4674	4674	4674	4674
Nek11	4674 / 4713	4674	4674	4674	4674
Nek2	4713	4674	4674	4674	4674
Nek4	4713	4674	4674	4674	4674
Nek6	4674 / 4713	4674	4674	4674	4674
Nek7	4674	4674	4674	4674	4674
Nlk	4674 / 4713	4674	4674	4674	4674
Npr1	4674 / 4713	4674	4713	4713	4713
Oxsr1	4674 / 4713	4674	4674	4674	4674
Pak1	4674 / 4713	4674	4674	4674	4674
Pak2	4674	4674	4674	4674	4674
Pak3	4713	4674	4674	4674	4674
Pak4	4674 / 4713	4674	4674	4674	4674
Pak7	4674 / 4713	4674	4674	4674	4674
Pask	4674 / 4713	4674	4674	4674	4674
Pbk	4674 / 4713	4674	4674	4674	4674
Pctk1	4713	4674	4674	4674	4674
Pctk3	4713	4674	4674	4674	4674
Pdgfra	4674	4713	4713	4713	4713
Pdgfrb	4674	4713	4713	4713	4713
Pdpk1	4674	4674 / 4713	4674	4674	4674
Pftk1	4674 / 4713	4674	4674	4674	4674
Phkg1	4713	4674	4674	4674	4674
Pim1	4713	4674	4674	4674	4674
Pim2	4674 / 4713	4674	4674	4674	4674
Pink1	4713	4674	4674	4674	4674
Pkmyt1	4713	4674 / 4713	4674	4674	4674
Pkn2	4674 / 4713	4674	4674	4674	4674
Plk1	4713	4674	4674	4674	4674
Plk2	4713	4674	4674	4674	4674
Plk4	4713	4674	4674	4674	4674
Pnck	4713	4674	4674	4674	4674
Prkaca	4674 / 4713	4674	4674	4674	4674
Prkca	4674 / 4713	4674	4674	4674	4674
Prkcb1	4674 / 4713	4674	4674	4674	4674
Prkcc	4674 / 4713	4674	4674	4674	4674
Prkch	4713	4674	4674	4674	4674
Prkci	4713	4674	4674	4674	4674
Prkcm	4713	4674	4674	4674	4674
Prkcz	4713	4674	4674	4674	4674
Prkg2	4713	4674	4674	4674	4674
Prkx	4674 / 4713	4674	4674	4674	4674
Prpf4b	4713	4674	4674	4674	4674
Ptk2	4674	4674	4713	4713	4713
Ptk6	4674	4713	4713	4713	4713
Pxk	4674	4674	4674	4674	4674

Ret	4674 / 4713	4713	4713	4713	4713
Ripk1	4713	4674	4674	4674	4674
Ripk5	4674 / 4713	4674	4674	4674	4674
Rock1	4713	4674	4674	4674	4674
Ror1	4674 / 4713	4713	4713	4713	4713
Ror2	4674 / 4713	4713	4713	4713	4713
Rps6ka1	4713	4674	4674	4674	4674
Rps6ka3	4674	4674	4674	4674	4674
Rps6ka5	4674 / 4713	4674	4674	4674	4674
Rps6kb2	4713	4674	4674	4674	4674
Rps6kl1	4674	4674	4674	4674	4674
Sbk1	4674 / 4713	4674	4674	4674	4674
Sgk2	4713	4674	4674	4674	4674
Sgk3	4674	4674	4674	4674	4674
Slk	4713	4674	4674	4674	4674
Snf1lk2	4674	4674	4674	4674	4674
Snrk	4674 / 4713	4674	4674	4674	4674
Src	4674 / 4713	4713	4713	4713	4713
Srpk1	4713	4674	4674	4674	4674
Srpk2	4713	4674	4674	4674	4674
Stk10	4674 / 4713	4674	4674	4674	4674
Stk16	4713	4674	4674	4674	4674
Stk17b	4674 / 4713	4674	4674	4674	4674
Stk23	4674	4674	4674	4674	4674
Stk32b	4713	4674	4674	4674	4674
Stk36	4674 / 4713	4674	4674	4674	4674
Stk38l	4674	4674	4674	4674	4674
Syk	4674 / 4713	4713	4713	4713	4713
Tbk1	4674 / 4713	4674	4674	4674	4674
Tec	4674	4713	4713	4713	4713
Tek	4674	4713	4713	4713	4713
Tgfbr1	4713	4674	4674	4674	4674
Tgfbr2	4713	4674	4674	4674	4674
Tie1	4674	4713	4713	4713	4713
Tlk1	4674 / 4713	4674	4674	4674	4674
Tlk2	4713	4674	4674	4674	4674
Tnk1	4674	4713	4713	4713	4713
Tnk2	4674 / 4713	4713	4713	4713	4713
Tssk1	4713	4674	4674	4674	4674
Tssk2	4713	4674	4674	4674	4674
Tssk6	4674 / 4713	4674	4674	4674	4674
Ttbk2	4674	4674	4674	4674	4674
Txk	4674	4713	4713	4713	4713
Tyk2	4674	4713	4713	4713	4713
Tyro3	4674	4713	4713	4713	4713
Vrk1	4674	4674	4674	4674	4674
Vrk2	4674	4674	4674	4674	4674
Vrk3	4674	4674	4674	4674	4674
Yes1	4674	4713	4713	4713	4713
Zap70	4713	4713	4713	4713	4713

Legend for Supplementary Table 3:

AmiGO labels, UniProt labels, and Predicted Labels for each mouse kinase protein

For the 244 mouse protein kinase used in this study, each row in the table contains the AmiGO annotation, UniProt annotation and predictions made by each of the three machine learning classifiers tested. These predictions are based on using a classifier built on 330 human proteins with protein gene ontology functional labels from AmiGO and verified by UniProt. Because the training data are fixed, note that the predictions will be the same regardless of what labels we use on the test set for evaluation purposes.

The ***Mouse Gene ID*** was obtained from each of the AmiGO protein records. The *AmiGO Label* field is “4713” (Tyr) if a query in AmiGO for the GO label GO0004713 returns the corresponding protein for mouse proteins, “4674” (Ser/Thr) if a query in AmiGO for the GO label GO0004674 returns the corresponding protein for mouse proteins, or “4674 / 4713” if a query in AmiGO for both GO labels GO0004674 and GO0004713 returns the corresponding protein. The ***UniProt Label*** field is “ 4713” if a search in UniProt with the AmiGO Gene ID returns a mouse protein that contains a reference to the functional class protein-tyrosine kinase activity, ”4674” if a search in UniProt with the AmiGO Gene ID returns a mouse protein that contains a reference to the functional class serine/threonine kinase activity, or “4674 / 4713” if a search in UniProt returns a mouse protein that contains a reference to the functional class serine/threonine kinase activity and protein-tyrosine kinase activity or any evidence that would suggest dual specificity. The ***Prediction of classifier #1*** field contains the prediction of the first HDTree classifier that distinguishes between GO0004674 and not GO0004674 (GO0004713 and Dual). This classifier was built on human proteins. The ***Prediction of classifier #2*** field contains the prediction of the second HDTree classifier that distinguishes between GO0004713 and not GO0004713 (GO0004674 and Dual). This classifier was built on human proteins. The ***Prediction of classifier #3*** field contains the prediction of the third classifier that distinguishes between GO0004674, GO0004713 and Dual. The third classifier combines the outputs of the first two classifiers to distinguish between GO0004674 and GO0004713 and Dual. (See **Supplementary Data** for details)
