# Supplementary material for: Exploring inconsistencies in genome-wide protein function annotations: a machine learning approach
Source: BMC Bioinformatics. 2007 Aug 3;8:284. doi: 10.1186/1471-2105-8-284 (PMC1994202; doi:10.1186/1471-2105-8-284)
Supplement: Additional file 4 — Supplementary Data: Machine learning approaches to predict Gene Ontology and/or UniProt Functional labels. The data provided represent the results and performance of all the machine learning approaches used in this study. [file 1471-2105-8-284-S4.pdf]

Supplementary Data: Machine Learning Results

Machine Learning approaches to predict Gene Ontology and/or UniProt Functional labels:

Experiments #1 - #7 show the results of several machine learning approaches used in this study. After the summary of each experiment, five sections summarize the performance obtained in each experiment. The first section displays the number of correctly and incorrectly classified instances along with the percent accuracy and percent error for each of the three classifiers (See **Methods** for details on each of the classifiers). The section summarizes the correlation coefficients and kappa coefficients for each of the methods. The third, fourth, and fifth sections show the individual performance of each of the three classifiers. Each row represents the functional class provided by the data label source (either AmiGO or UniProt). Each column represents the functional class *predicted* by the given classifier. The far right column shows the Recall for each of the classes and the last row shows the Precision for each of the classes (in **Methods**). The accuracy of the classifier can be found where the Recall column and Precision row intersect.

Experiment #1: Human cross-validation Summary

Training Method: 10-fold cross-validation
Species: Human
Evidence Code: All
Data Label Source: AmiGO
Total Number of Instances: 330

	Classifier #1		Classifier #2		**Classifier #3**	
	Instances	Percent	Instances	Percent	**Instances**	**Percent**
Correctly Classified	302	91.5%	316	95.8%	**294**	**89.1%**
Incorrectly Classified	28	8.5%	14	4.2%	**36**	**10.9%**

	Correlation Coefficient			Kappa Coefficient
	Ser/Thr	Tyr	Dual	
Classifier #1	0.79	--	--	0.79
Classifier #2	--	0.89	--	0.89
Classifier #3	0.82	0.86	0.30	0.76

=== Classifier #1 Overall Performance Evaluation ===

classified as →	GO0004674 (Ser/Thr)	GO0004713 (Tyr) and Dual	Recall
GO0004674(Ser/Thr)	227	6	0.97
GO0004713(Tyr) and Dual	22	75	0.77
Precision →	0.91	0.93	Accuracy = 0.92

=== Classifier #2 Overall Performance Evaluation ===

classified as →	GO0004713 (Tyr)	GO0004674 (Ser/Thr) and Dual	Recall
GO0004713(Tyr)	81	9	0.90
GO0004674(Ser/Thr) and Dual	5	235	0.98
Precision →	0.94	0.96	Accuracy = 0.96

=== Classifier #3 Overall Performance Evaluation ===

classified as →	GO0004713 (Tyr)	GO0004674 (Ser/Thr)	Dual	Recall
GO0004713 (Tyr)	67	6	17	0.74
GO0004674 (Ser/Thr)	0	222	11	0.95
Dual	0	2	5	0.71
Precision →	1.00	0.97	0.15	Accuracy = 0.89

Experiment #2: Human Training Set / Mouse Test Set Summary

**Training Method:**	Trained on Human / Tested on Mouse
**Species:**	Human (Training) / Mouse (Testing)
**Evidence Code:**	All
**Data Label Source:**	AmiGO
**Total Number of Instances:**	330 (Training) / 244 (Testing)

	Classifier #1		Classifier #2		Classifier #3	
	Instances	Percent	Instances	Percent	Instances	Percent
Correctly Classified	57	23.4%	86	35.2%	37	15.1%
Incorrectly Classified	187	76.6%	158	64.8%	207	84.9%

	Correlation Coefficient			Kappa Coefficient
	Ser/Thr	Tyr	Dual	
Classifier #1	-0.43	--	--	-0.30
Classifier #2	--	-0.40	--	-0.38
Classifier #3	-0.40	-0.43	-0.01	-0.40

=== Classifier #1 Overall Performance Evaluation ===

classified as →	GO0004674 (Ser/Thr)	GO0004713 (Tyr) and Dual	Recall
GO0004674(Ser/Thr)	29	42	0.41
GO0004713(Tyr) and Dual	145	28	0.16
Precision →	0.17	0.40	Accuracy = 0.23

=== Classifier #2 Overall Performance Evaluation ===

classified as →	GO0004713 (Tyr)	GO0004674 (Ser/Thr) and Dual	Recall
GO0004713(Tyr)	7	99	0.07
GO0004674(Ser/Thr) and Dual	59	79	0.57
Precision →	0.11	0.44	Accuracy = 0.35

=== Classifier #3 Overall Performance Evaluation ===

classified as →	GO0004713 (Tyr)	GO0004674 (Ser/Thr)	Dual	Recall
GO0004713 (Tyr)	7	96	3	0.07
GO0004674 (Ser/Thr)	42	29	0	0.41
Dual	17	49	1	0.01
Precision →	0.11	0.17	0.25	Accuracy = 0.15

Experiment #3: Human Training Set/ Mouse Test Set (RCA only) Summary

**Training Method:**	Trained on Human / Tested on Mouse
**Species:**	Human (Training) / Mouse (Testing)
**Evidence Code:**	RCA (inferred from Reviewed Computational Analysis)
**Data Label Source:**	AmiGO
**Total Number of Instances:**	330 (Training) / 211 (Testing)

	Classifier #1		Classifier #2		Classifier #3	
	Instances	Percent	Instances	Percent	Instances	Percent
Correctly Classified	13	6.2%	43	20.4%	9	4.2%
Incorrectly Classified	198	93.8%	168	79.6%	202	95.8%

	Correlation Coefficient			Kappa Coefficient
	Ser/Thr	Tyr	Dual	
Classifier #1	-0.85	--	--	-0.60
Classifier #2	--	-0.64	--	-0.57
Classifier #3	-0.64	-0.85	0.00	0.50

=== Classifier #1 Overall Performance Evaluation ===

classified as →	GO0004674 (Ser/Thr)	GO0004713 (Tyr) and Dual	Recall
GO0004674(Ser/Thr)	9	55	0.14
GO0004713(Tyr) and Dual	143	4	0.03
Precision →	0.06	0.07	Accuracy = 0.06

=== Classifier #2 Overall Performance Evaluation ===

classified as →	GO0004713 (Tyr)	GO0004674 (Ser/Thr) and Dual	Recall
GO0004713(Tyr)	0	109	0.00
GO0004674(Ser/Thr) and Dual	59	43	0.42
Precision →	0.00	0.28	Accuracy = 0.20

=== Classifier #3 Overall Performance Evaluation ===

classified as →	GO0004713 (Tyr)	GO0004674 (Ser/Thr)	Dual	Recall
GO0004713 (Tyr)	0	109	0	0.00
GO0004674 (Ser/Thr)	55	9	0	0.14
Dual	4	34	0	0.00
Precision →	0.00	0.06	0.00	Accuracy = 0.04

Experiment #4: Human Training Set / Mouse Test Set (at least one RCA code) Summary

**Training Method:**	Trained on Human / Tested on Mouse
**Species:**	Human (Training) / Mouse (Testing)
**Evidence Code:**	at least one RCA evidence code (but also including other evidence codes in the annotation)
**Data Label Source:**	AmiGO
**Total Number of Instances:**	330 (Training) / 211 (Testing)

	Classifier #1		Classifier #2		Classifier #3	
	Instances	Percent	Instances	Percent	Instances	Percent
Correctly Classified	26	12.3%	58	27.5%	9	4.2%
Incorrectly Classified	185	87.7%	153	72.5%	202	95.8%

	Correlation Coefficient			Kappa Coefficient
	Ser/Thr	Tyr	Dual	
Classifier #1	-0.68	--	--	-0.42
Classifier #2	--	-0.56	--	-0.52
Classifier #3	-0.56	-0.68	0.00	-0.37

=== Classifier #1 Overall Performance Evaluation ===

classified as →	GO0004674 (Ser/Thr)	GO0004713 (Tyr) and Dual	Recall
GO0004674(Ser/Thr)	9	42	0.18
GO0004713(Tyr) and Dual	143	17	0.11
Precision →	0.06	0.29	Accuracy = 0.12

=== Classifier #2 Overall Performance Evaluation ===

classified as →	GO0004713 (Tyr)	GO0004674 (Ser/Thr) and Dual	Recall
GO0004713(Tyr)	0	94	0.00
GO0004674(Ser/Thr) and Dual	59	58	0.50
Precision →	0.00	0.38	Accuracy = 0.27

=== Classifier #3 Overall Performance Evaluation ===

classified as →	GO0004713 (Tyr)	GO0004674 (Ser/Thr)	Dual	Recall
GO0004713 (Tyr)	0	94	0	0.00
GO0004674 (Ser/Thr)	42	9	0	0.18
Dual	17	49	0	0.00
Precision →	0.00	0.06	0.00	Accuracy = 0.04

Experiment #5: Human Training Set / Mouse Test Set (UniProt label) Summary

Training Method: Trained on Human / Tested on Mouse
Species: Human (Training) / Mouse (Testing)
Evidence Code: All
Data Label Source: UniProt
Total Number of Instances: 330 (Training) / 244 (Testing)

	Classifier #1		Classifier #2		Classifier #3	
	Instances	Percent	Instances	Percent	Instances	Percent
Correctly Classified	234	95.9%	241	98.8%	233	95.4%
Incorrectly Classified	10	4.1%	3	1.2%	11	4.6%

	Correlation Coefficient			Kappa Coefficient
	Ser/Thr	Tyr	Dual	
Classifier #1	0.90	--	--	0.90
Classifier #2	--	0.97	--	0.97
Classifier #3	0.96	0.90	0.43	0.90

=== Classifier #1 Overall Performance Evaluation ===

classified as →	GO0004674 (Ser/Thr)	GO0004713 (Tyr) and Dual	Recall
GO0004674(Ser/Thr)	166	2	0.99
GO0004713(Tyr) and Dual	8	68	0.89
Precision →	0.95	0.97	Accuracy = 0.96

=== Classifier #2 Overall Performance Evaluation ===

classified as →	GO0004713 (Tyr)	GO0004674 (Ser/Thr) and Dual	Recall
GO0004713(Tyr)	64	1	0.98
GO0004674(Ser/Thr) and Dual	2	177	0.99
Precision →	0.97	0.99	Accuracy = 0.99

=== Classifier #3 Overall Performance Evaluation ===

classified as →	GO0004713 (Tyr)	GO0004674 (Ser/Thr)	Dual	Recall
GO0004713 (Tyr)	64	0	1	0.98
GO0004674 (Ser/Thr)	2	166	0	0.99
Dual	0	8	3	0.27
Precision →	0.97	0.95	0.75	Accuracy = 0.95

Experiment #6: Human Training Set / Mouse Test Set (RCA only/UniProt label) Summary

**Training Method:**	Trained on Human / Tested on Mouse
**Species:**	Human (Training) / Mouse (Testing)
**Evidence Code:**	All
**Data Label Source:**	UniProt
**Total Number of Instances:**	330 (Training) / 211 (Testing)

	Classifier #1		Classifier #2		Classifier #3	
	Instances	Percent	Instances	Percent	Instances	Percent
Correctly Classified	205	97.1%	209	99.1%	205	97.1%
Incorrectly Classified	6	2.9%	2	0.9%	6	2.9%

	Correlation Coefficient			Kappa Coefficient
	Ser/Thr	Tyr	Dual	
Classifier #1	0.93	--	--	0.93
Classifier #2	--	0.98	--	0.98
Classifier #3	0.98	0.94	0.00	0.93

=== Classifier #1 Overall Performance Evaluation ===

classified as →	GO0004674 (Ser/Thr)	GO0004713 (Tyr) and Dual	Recall
GO0004674(Ser/Thr)	148	2	0.99
GO0004713(Tyr) and Dual	4	57	0.93
Precision →	0.97	0.97	Accuracy = 0.97

=== Classifier #2 Overall Performance Evaluation ===

classified as →	GO0004713 (Tyr)	GO0004674 (Ser/Thr) and Dual	Recall
GO0004713(Tyr)	57	0	1.00
GO0004674(Ser/Thr) and Dual	2	152	0.99
Precision →	0.97	1.00	Accuracy = 0.99

=== Classifier #3 Overall Performance Evaluation ===

classified as →	GO0004713 (Tyr)	GO0004674 (Ser/Thr)	Dual	Recall
GO0004713 (Tyr)	57	0	0	1.00
GO0004674 (Ser/Thr)	2	148	0	0.99
Dual	0	4	0	0.00
Precision →	0.97	0.97	0.00	Accuracy = 0.97

Experiment #7: Human Training Set / Mouse Test Set (at least one RCA code/UniProt label)*:

* Please note that these results were identical to Experiment #6 results since the evaluation is based on UniProt labels and not the labels given by AmiGO.

Comparing AmiGO and UniProt Labels:

Experiments #8 - #10 show tables corresponding to the number of proteins with the given functional labels given by AmiGO and by UniProt. These data were taken directly from each database; no machine learning approaches were used. Columns represent the number of proteins retrieved by AmiGO that had the corresponding Column header as a functional label given by AmiGO. Rows represent the number of proteins retrieved by AmiGO that had the corresponding Row header as functional evidence within UniProt.

Experiment #8: Mouse Data (All): UniProt versus AmiGO

UniProt Labels	GO0004713 (Tyr)	GO0004674 (Ser/Thr)	Dual	UniProt Total
GO0004713 (Tyr)	8	41	16	65
GO0004674 (Ser/Thr)	91	28	49	168
Dual	7	2	2	11
AmiGO Total →	106	71	67	Total # proteins =244

Instances in Agreement:	38	15.6%
Instances in Disagreement:	206	84.4%

Experiment #9: Mouse Data (RCA evidence code only): UniProt versus AmiGO

UniProt Labels	GO0004713 (Tyr)	GO0004674 (Ser/Thr)	Dual	UniProt Total
GO0004713 (Tyr)	0	54	3	57
GO0004674 (Ser/Thr)	105	10	35	150
Dual	4	0	0	4
AmiGO Total →	109	64	38	Total # proteins =211

Instances in Agreement:	10	4.7%
Instances in Disagreement:	201	95.3%

Experiment #10: Mouse Data (at least one RCA evidence code): UniProt versus AmiGO

UniProt Labels	GO0004713 (Tyr)	GO0004674 (Ser/Thr)	Dual	UniProt Total
GO0004713 (Tyr)	0	41	16	57
GO0004674 (Ser/Thr)	91	10	49	150
Dual	3	0	1	4
AmiGO Total →	94	51	66	Total # proteins =211

Instances in Agreement:	11	5.2%
Instances in Disagreement:	200	94.8%
