# Supplementary material for: Exploring inconsistencies in genome-wide protein function annotations: a machine learning approach
Source: BMC Bioinformatics. 2007 Aug 3;8:284. doi: 10.1186/1471-2105-8-284 (PMC1994202; doi:10.1186/1471-2105-8-284)
Supplement: Additional file 5 — Supplementary Table 4: Mouse kinases having a human ortholog. A table displaying the human orthologs for the mouse kinases used in this study. The table also displays the identity between these orthologs. [file 1471-2105-8-284-S5.pdf]

Supplementary Table 4:

Mouse Kinases having a Human Ortholog (See Table legend below)

Gene ID (AmiGO)	Jackson Lab Symbol (Mouse Kinome)	Gene ID (Mouse Kinome)	Human Ortholog (Human Kinome)	Identity between orthologs
2610018G03Rik				
Acvr1b	Acvr1b	ALK4	ALK4	99.31%
Acvr2a				
Acvr2b	Acvr2b	ACTR2B	ACTR2B	100.00%
Acvrl1				
Adrbk1	Adrbk1	BARK1	BARK1	99.62%
Akt1	Akt1	AKT1	AKT1	99.61%
Alk	Alk	ALK	ALK	98.15%
Araf	Araf	ARAF	ARAF	98.45%
Atm	Atm	ATM	ATM	N/A
Aurka				
Aurkb				
Axl	Axl	AXL	AXL	97.40%
Blk	Blk	BLK	BLK	93.63%
Bmpr1a	Bmpr1a	BMPR1A	BMPR1A	99.65%
Bmpr1b	Bmpr1b	BMPR1B	BMPR1B	98.96%
Bmpr2	Bmpr2	BMPR2	BMPR2	98.70%
Bmx	Bmx	BMX	BMX	94.44%
Btk	Btk	BTK	BTK	98.81%
Camk1				
Camk1d	E030025C11Rik	CAMK1d	CAMK1d	100.00%
Camk1g	Camk1g	CAMK1g	CAMK1g	98.43%
Camk2a	Camk2a	CaMK2a	CaMK2a	100.00%
Camk2b	Camk2b	CaMK2b	CaMK2b	100.00%
Camk2g	Camk2g	CaMK2g	CaMK2g	100.00%
Camk4	Camk4	CaMK4	CaMK4	99.61%
Camkk1	Camkk1	CAMKK1	CAMKK1	93.45%
Ccrk	4932702G04Rik	CCRK	CCRK	94.04%
Cdc2a	Cdc2a	CDC2	CDC2	97.18%
Cdc2l5				
Cdk5	Cdk5	CDK5	CDK5	99.65%
Cdk7	Cdk7	CDK7	CDK7	97.18%
Cdk9	Cdk9	CDK9	CDK9	98.32%
Cdkl1	Cdkl1	CDKL1	CDKL1	95.42%
Cdkl3	Cdkl3	CDKL3	CDKL3	93.29%
Cdkl4	AU067824	CDKL4	CDKL4	91.87%
Chek1	Chek1	CHK1	CHK1	96.89%
Chek2	Chek2	CHK2	CHK2	92.13%
Chuk	Chuk	IKKa	IKKa	97.08%

Cit				
Clk1	Clk	CLK1	CLK1	94.32%
Clk2	Clk2	CLK2	CLK2	98.42%
Clk3		CLK3	CLK3	100.00%
Clk4	Clk4	CLK4	CLK4	98.73%
Cpne3				
Csf1r				
Csk	Csk	CSK	CSK	99.19%
Csnk1d	Csnk1d	CK1d	CK1d	100.00%
Csnk1e	Csnk1e	CK1e	CK1e	100.00%
Csnk1g2				
Csnk2a2	Csnk2a2	CK2a2	CK2a2	99.30%
Dapk2	Dapk2	DAPK2	DAPK2	98.86%
Dapk3	Dapk3	DAPK3	DAPK3	95.44%
Dcamkl2	6330415M09Rik	DCAMKL2	DCAMKL2	97.29%
Ddr1	Ddr1	DDR1	DDR1	92.91%
Dmpk	Dm15	DMPK1	DMPK1	93.31%
Dyrk1a	Dyrk1a	DYRK1A	DYRK1A	99.69%
Egfr	Egfr	EGFR	EGFR	98.84%
Eif2ak1				
Eif2ak3				
Eif2ak4				
Epha1	Epha1	EphA1	EphA1	93.85%
Epha2	Epha2	EphA2	EphA2	96.12%
Epha3	Mark3	EphA3	EphA3	100.00%
Epha4	Epha4	EphA4	EphA4	100.00%
Epha5	Epha5	EphA5	EphA5	98.83%
Epha6	Epha6	EPHA6	EPHA6	98.67%
Epha7	Epha7	EphA7	EphA7	99.61%
Epha8	Epha8	EphA8	EphA8	93.41%
Ephb2	Ephb2	EphB2	EphB2	100.00%
Ephb3	Ephb3	EphB3	EphB3	99.62%
Ephb4	Ephb4	EphB4	EphB4	99.65%
Ephb6	Ephb6	EphB6	EphB6	93.90%
Erbb2	Erbb2	ErbB2	ErbB2	98.45%
Ern2				
Fgfr1	Fgfr1	FGFR1	FGFR1	100.00%
Fgfr2	Fgfr2	FGFR2	FGFR2	99.64%
Fgfr3	Fgfr4	FGFR4	FGFR4	97.47%
Fgfr4	Fgfr4	FGFR4	FGFR4	97.47%
Fgr	Fgr	FGR	FGR	92.00%
Flt1	Flt1	FLT1	FLT1	91.46%
Flt3	Flt3	FLT3	FLT3	93.11%
Flt4	Flt4	FLT4	FLT4	91.69%

Fyn	Fyn	FYN	FYN	99.20%
Gprk2l	Gprk2l	GPRK4	GPRK4	85.55%
Gprk5	Gprk5	GPRK5	GPRK5	98.86%
Gprk6	Gprk6	GPRK6	GPRK6	97.34%
Grk1				
Gsg2				
Gsk3b	Gsk3b	GSK3B	GSK3B	100.00%
Hck	Hck	HCK	HCK	94.42%
Hipk2	Hipk2	HIPK2	HIPK2	99.39%
Hipk3	Hipk3	HIPK3	HIPK3	97.87%
Hunk	Hunk	HUNK	HUNK	98.08%
Ick	Ick	ICK	ICK	97.15%
Igf1r	Igf1r	IGF1R	IGF1R	97.41%
Ikbkb	Ikbkb	IKKb	IKKb	96.73%
Ikbke	Ikbke	IKKe	IKKe	91.51%
Ilk	Taf10	ILK	ILK	99.22%
Insrr	Insrr	IRR	IRR	93.70%
Irak3	Irak3	IRAK3	IRAK3	83.15%
Itk	Itk	ITK	ITK	96.80%
Jak1	Jak1	JAK1	JAK1	97.83%
Jak2	Jak2	JAK2	JAK2	97.12%
Jak3	Jak3	JAK3	JAK3	82.72%
Kdr	Kdr	KDR	KDR	96.33%
Kit	Kit	KIT	KIT	94.94%
Ksr1	Ksr	KSR1	KSR1	95.83%
Lats1	Lats1	LATS1	LATS1	98.69%
Lck	Lck	LCK	LCK	97.21%
Limk1	Limk1	LIMK1	LIMK1	97.37%
Lrrk1	D130026O16Rik	LRRK1	LRRK1	94.37%
Ltk	Ltk	LTK	LTK	88.89%
Lyn	Lyn	LYN	LYN	97.62%
Map2k3	Map2k3	MAP2K3	MAP2K3	98.09%
Map2k5	Map2k5	MAP2K5	MAP2K5	98.36%
Map3k12				
Map3k14				
Map3k3	Map3k3	MAP3K3	MAP3K3	99.23%
Map3k4	Map3k4	MAP3K4	MAP3K4	98.07%
Map3k7		MAP3K7	MAP3K7	96.47%
Map3k8		MAP3K8	MAP3K8	94.70%
Map4k1				
Map4k2				
Mapk1				
Mapk10				
Mapk11				

Mapk12				
Mapk13				
Mapk14				
Mapk3				
Mapk7				
Mapk8				
Mapk9				
Mapkapk2	Mapkapk2	MAPKAPK2	MAPKAPK2	98.47%
Mapkapk5	Mapkapk5	MAPKAPK5	MAPKAPK5	98.59%
Mark1	B930025N23Rik	MARK1	MARK1	99.21%
Mark2	Mark2	MARK2	MARK2	100.00%
Mast1	Mast1	MAST1	MAST1	99.64%
Mast2	Mtssk	MAST2	MAST2	98.91%
Mastl	2700091H24Rik	MASTL	MASTL	87.32%
Matk				
Melk	Melk	MELK	MELK	95.26%
Mertk	Mertk	MER	MER	93.61%
Met	Met	MET	MET	97.71%
Mknk1				
Mos		MOS	MOS	75.09%
Musk	Musk	MUSK	MUSK	97.15%
Mylk2				
Nek11				
Nek2	Nek2	NEK2	NEK2	93.56%
Nek4	Nek4	NEK4	NEK4	96.48%
Nek6	Nek6	NEK6	NEK6	98.41%
Nek7	Nek7	NEK7	NEK7	98.41%
Nlk	Nlk	NLK	NLK	100.00%
Npr1				
Oxsr1				
Pak1	Pak1	PAK1	PAK1	99.60%
Pak2	Pak2	PAK2	PAK2	99.60%
Pak3	Pak3	PAK3	PAK3	98.81%
Pak4	Pak4	PAK4	PAK4	98.41%
Pak7				
Pask	Pask	PASK	PASK	88.93%
Pbk	Topk-pending	PBK	PBK	89.55%
Pctk1				
Pctk3				
Pdgfra	Pdgfra	PDGFRa	PDGFRa	98.32%
Pdgfrb	Pdgfrb	PDGFRb	PDGFRb	94.71%
Pdpk1				
Pftk1				
Phkg1	Phkg	PHKg1	PHKg1	93.31%
Pim1	Pim1	PIM1	PIM1	95.26%

Pim2	Pim2	PIM2	PIM2	92.16%
Pink1	1190006F07Rik	PINK1	PINK1	81.25%
Pkmyt1				
Pkn2				
Plk1	Plk	PLK1	PLK1	96.84%
Plk2	Snk	PLK2	PLK2	99.60%
Plk4	Stk18	PLK4	PLK4	95.67%
Pnck				
Prkaca				
Prkca	Prkca	PKCa	PKCa	100.00%
Prkcb1				
Prkcc				
Prkch				
Prkci				
Prkcm				
Prkcz				
Prkg2				
Prkx				
Prpf4b				
Ptk2				
Ptk6				
Pxk				
Ret	Ret	RET	RET	95.42%
Ripk1	Ripk1	RIPK1	RIPK1	76.58%
Ripk5				
Rock1	Rock1	ROCK1	ROCK1	100.00%
Ror1	Ror1	ROR1	ROR1	98.70%
Ror2	Ror2	ROR2	ROR2	96.09%
Rps6ka1				
Rps6ka3				
Rps6ka5				
Rps6kb2				
Rps6kl1				
Sbk1				
Sgk2	Sgk2	SGK2	SGK2	94.57%
Sgk3	Sgk3	SGK3	SGK3	98.45%
Slk	Stk2	SLK	SLK	98.46%
Snf1lk2				
Snrk	Snrk	SNRK	SNRK	95.67%
Src	Src	SRC	SRC	99.20%
Srpk1	Srpk1	SRPK1	SRPK1	90.59%
Srpk2	Srpk2	SRPK2	SRPK2	93.73%
Stk10				
Stk16				
Stk17b				
Stk23				
Stk32b				

Stk36				
Stk38l				
Syk	Syk	SYK	SYK	99.20%
Tbk1	Tbk1	TBK1	TBK1	97.97%
Tec	Tec	TEC	TEC	96.00%
Tek	Tek	TIE2	TIE2	99.63%
Tgfbr1	Tgfbr1	TGFBR1	TGFBR1	100.00%
Tgfbr2	Tgfbr2	TGFBR2	TGFBR2	98.31%
Tie1	Tie1	TIE1	TIE1	99.63%
Tlk1	Tlk1	TLK1	TLK1	100.00%
Tlk2	Tlk2	TLK2	TLK2	99.64%
Tnk1	Tnk1	TNK1	TNK1	93.51%
Tnk2				
Tssk1	Stk22a	TSSK1	TSSK1	92.34%
Tssk2	Stk22b	TSSK2	TSSK2	94.64%
Tssk6				
Ttbk2	B930008N24Rik	TTBK2	TTBK2	98.44%
Txk	Txk	TXK	TXK	87.70%
Tyk2	Tyk2	TYK2	TYK2	91.73%
Tyro3	Tyro3	TYRO3	TYRO3	98.85%
Vrk1	Vrk1	VRK1	VRK1	93.63%
Vrk2	Vrk2	VRK2	VRK2	84.85%
Vrk3	AI428238	VRK3	VRK3	81.25%
Yes1				
Zap70	Zap70	ZAP70	ZAP70	95.60%

Legend for Supplementary Table 4:

Mouse Kinases having a Human Ortholog

All 244 mouse protein kinases used in this study were compared to the mouse proteins found in the Mouse Kinome. If a match in the Kinome database [1-3] was found the corresponding human ortholog and its sequence identity to the mouse protein are displayed in the table (167 proteins in total). If a match was not found, the row in the table is left blank (77 proteins in total). To match up the AmiGO proteins with the proteins found in Mouse Kinome [1] we used the ***Mouse Gene ID*** obtained from the AmiGO record and the ***Jackson Lab Symbol*** found in the second table of the Mouse Kinome [1-3]. The Mouse Kinome database directly provided the ***Human Ortholog ID*** and the percent sequence identity between the Mouse and Human orthologs (***Percent Identity between Orthologs*** field above). We did not compute this identity directly for this study. A brief summary of this table can be found in **Supplementary Table 5**.

References for Supplementary Table 4:

1. Caenepeel, S., Charydczak, G., Sudarsanam, S., Hunter, T. & Manning, G. *PNAS* **101**, 11707-11712. (2004). http://kinase.com/mouse/
2. Manning, G., Whyte, D.B., Martinez, R., Hunter, T., & Sudarsanam, S. *Science* **298**, 1912-1934 (2002). http://kinase.com/human/kinome/
3. http://kinase.com/mouse/tables/Table2.xls
