# Supplementary material for: Exploring inconsistencies in genome-wide protein function annotations: a machine learning approach
Source: BMC Bioinformatics. 2007 Aug 3;8:284. doi: 10.1186/1471-2105-8-284 (PMC1994202; doi:10.1186/1471-2105-8-284)
Supplement: Additional file 6 — Supplementary Table 5: Number of mouse kinases having a specified level of sequence identity with their human orthologs. A table displaying the summary statistics of Supplementary Table 4. [file 1471-2105-8-284-S6.pdf]

Supplementary Table 5:

Sequence Identity cutoff with Human Ortholog	Number of mouse proteins	Percent of 167 mouse kinases with Human Ortholog	Percent of all 244 mouse kinases
100%	19	11.4%	7.8%
99%	46	27.5%	18.9%
95%	118	70.7%	48.4%
90%	154	92.2%	63.1%
85%	160	95.8%	65.6%
80%	165	98.8%	67.6%
75%	167	100%	68.4%

Legend for Supplementary Table 5:

Number of Mouse kinases having a specified level of sequence identity with their human orthologs. (Summary statistics for Supplementary Table 4).

We compared the sequence identities between each of the 244 mouse protein kinases used in this study with their human orthologs found in the Mouse Kinome (See **Supplementary Table 4)**. This table summarizes the number of proteins that had sequence identities greater than a fixed cutoff value.
