# Supplementary material for: Exploring inconsistencies in genome-wide protein function annotations: a machine learning approach
Source: BMC Bioinformatics. 2007 Aug 3;8:284. doi: 10.1186/1471-2105-8-284 (PMC1994202; doi:10.1186/1471-2105-8-284)
Supplement: Additional file 7 — Supplementary Note. Because there is only a non-curated reference to the work done on "Rat ISS GO annotations from MGI's mouse gene data," we provide the abstract and a link to the original reference report in this file. [file 1471-2105-8-284-S7.pdf]

Supplementary Note:

Because there is only a non-curated reference to the work done on “Rat ISS GO annotations from MGI's mouse gene data,” we provide the abstract and a link to the original reference report. The Rat Genome Database ID for this report is 1578720. The original report was created on April, 6, 2006.

Abstract

All the annotations assigned to mouse genes by the Mouse Genome Database (http://www.informatics.jax.org/) were uploaded from the Gene Ontology Consortium website (http://www.geneontology.org/GO.current.annotations.shtml). Annotations with "IEA" or "ND" Evidence Codes were removed and the remainder loaded onto the corresponding orthologous rat genes with an evidence code for the annotation to the rat gene of ISS. The decision to use ISS for the evidence code was made following a personal communication between Dr. Susan Bromberg (RGD) and Dr. Judith Blake (MGI/GO Consortium).

Original link:

http://rgd.mcw.edu/tools/references/references_view.cgi?id=1578720
