# Supplementary material for: Exploring inconsistencies in genome-wide protein function annotations: a machine learning approach
Source: BMC Bioinformatics. 2007 Aug 3;8:284. doi: 10.1186/1471-2105-8-284 (PMC1994202; doi:10.1186/1471-2105-8-284)
Supplement: Additional file 8 — Supplementary Table 6: The UniProt and AmiGO annotations for the rat kinase proteins with mouse orthologs. This table displays the UniProt and AmiGO annotations for rat kinase proteins that were annotated based on a mouse ortholog. [file 1471-2105-8-284-S8.pdf]

Supplementary Table 6:

The UniProt and AmiGO annotations for the Rat kinase proteins with Mouse orthologs (See Table legend below)

Mouse / Rat Gene ID	Mouse AmiGO label	Rat AmiGO label by ISS Annotation	UniProt label for Mouse
Acvr2a	4713	4713	4674
Acvr2b	4713	4713	4674
Acvrl1	4713	4713	4674
Adrbk1	4713	4713	4674
Akt1	4713	4713	4674
Alk	4674	4674	4713
Araf	4713	4713	4674
Aurkb	4713	4713	4674
Axl	4674	4674	4713
Blk	4674	4674 / 4713	4713
Bmpr1a	4713	4713	4674
Bmpr2	4713	4713	4674
Btk	4674 / 4713	4674 / 4713	4713
Camk1	4713	4713	4674
Camk1g	4674	4674	4674
Camk2a	4674	4674	4674
Camk2b	4674 / 4713	4674 / 4713	4674
Camk2g	4674 / 4713	4674 / 4713	4674
Camkk1	4674 / 4713	4713	4674
Ccrk	4674 / 4713	4674 / 4713	4674
Cdc2a	4713	4713	4674
Cdc2l5	4674 / 4713	4713	4674
Cdk5	4674	4674	4674
Cdk7	4674 / 4713	4713	4674 / 4713
Cdk9	4713	4713	4674 / 4713
Cdkl1	4674 / 4713	4674 / 4713	4674
Cdkl3	4674 / 4713	4674 / 4713	4674
Chek1	4713	4713	4674
Chek2	4713	4713	4674
Chuk	4713	4713	4674
Clk1	4674 / 4713	4713	4674 / 4713
Clk3	4713	4713	4674 / 4713
Clk4	4713	4713	4674 / 4713
Csf1r	4674	4674	4713
Csk	4674	4674	4713
Csnk1d	4713	4713	4674
Csnk1e	4713	4713	4674
Csnk1g2	4713	4713	4674
Csnk2a2	4674 / 4713	4713	4674
Dapk2	4713	4713	4674
Dapk3	4713	4713	4674
Ddr1	4674	4713	4713
Dmpk	4713	4713	4674

Dyrk1a	4713	4713	4674 / 4713
Eif2ak1	4713	4713	4674
Eif2ak3	4713	4713	4674
Eif2ak4	4674 / 4713	4674 / 4713	4674
Epha1	4674 / 4713	4674	4713
Epha2	4674	4674	4713
Epha3	4674 / 4713	4674	4713
Epha5	4674	4674	4713
Epha6	4674	4674	4713
Epha7	4674	4674	4713
Epha8	4674	4674	4713
Ephb3	4674 / 4713	4674	4713
Ephb6	4674	4674	4713
Erbb2	4674 / 4713	4674	4713
Ern2	4713	4713	4674
Fgfr1	4674 / 4713	4674	4713
Fgfr2	4674	4674	4713
Fgfr4	4674	4674	4713
Fgr	4674	4674	4713
Flt1	4674	4674	4713
Flt3	4674	4674	4713
Flt4	4674	4674	4713
Fyn	4713	4713	4713
Gprk2l	4674 / 4713	4713	4674
Gprk5	4674 / 4713	4674 / 4713	4674
Gprk6	4713	4713	4674
Grk1	4713	4713	4674
Hck	4674	4674	4713
Hipk2	4674	4674	4674
Hipk3	4713	4713	4674
Ick	4674	4674	4674
Igf1r	4674	4674	4713
Ikbkb	4713	4713	4674
Ikbke	4674 / 4713	4713	4674
Ilk	4674	4674	4674
Irak3	4674 / 4713	4674 / 4713	4674
Itk	4674	4674 / 4713	4713
Jak1	4713	4713	4713
Jak2	4674 / 4713	4674 / 4713	4713
Jak3	4713	4713	4713
Kit	4674 / 4713	4674	4713
Ksr1	4713	4713	4674 / 4713
Lck	4674	4674	4713
Limk1	4713	4713	4674
Lyn	4713	4713	4713
Map2k3	4713	4713	4674
Map2k5	4713	4713	4674
Map3k12	4713	4713	4674
Map3k14	4713	4713	4674
Map3k3	4713	4713	4674

Map3k4	4713	4713	4674
Map3k7	4713	4713	4674
Map3k8	4713	4713	4674
Map4k1	4674 / 4713	BOTH	4674
Map4k2	4713	4713	4674
Mapk1	4674 / 4713	BOTH	4674
Mapk10	4713	4713	4674
Mapk11	4713	4713	4674
Mapk12	4713	4713	4674
Mapk13	4713	4713	4674
Mapk14	4713	4713	4674
Mapk3	4713	4713	4674
Mapk7	4713	4713	4674
Mapk8	4713	4713	4674
Mark1	4674 / 4713	4674 / 4713	4674
Mast2	4674 / 4713	4674 / 4713	4674
Mastl	4674	4674	4674
Matk	4674	4674	4713
Melk	4674 / 4713	4674 / 4713	4674
Met	4674 / 4713	4674	4713
Mylk2	4674	4674	4674
Nek11	4674 / 4713	4674 / 4713	4674
Nek6	4674 / 4713	4674 / 4713	4674
Nek7	4674	4674	4674
Npr1	4674 / 4713	4674	4674
Oxsr1	4674 / 4713	4674 / 4713	4674
Pak1	4674 / 4713	4674 / 4713	4674
Pak2	4674	4674	4674
Pak4	4674 / 4713	4674 / 4713	4674
Pak7	4674 / 4713	4713	4674
Pask	4674 / 4713	4674 / 4713	4674
Pbk	4674 / 4713	4674 / 4713	4674
Pdgfra	4674	4674	4713
Pdgfrb	4674	4674	4713
Pftk1	4674 / 4713	4674 / 4713	4674
Pkn2	4674 / 4713	4674 / 4713	4674
Prkca	4674 / 4713	4674 / 4713	4674
Prkcb1	4674 / 4713	4674 / 4713	4674
Prkcc	4674 / 4713	4674 / 4713	4674
Ptk2	4674	4674 / 4713	4674
Ptk6	4674	4674	4713
Pxk	4674	4674	4674
Rock1	4713	4713	4674
Ror2	4674 / 4713	4674	4713
Rps6ka1	4713	4713	4674
Rps6ka5	4674 / 4713	4674 / 4713	4674
Rps6kb2	4713	4713	4674
Sgk2	4713	4713	4674
Snrk	4674 / 4713	4713	4674
Src	4674 / 4713	4713	4713

Srpk1	4713	4713	4674
Srpk2	4713	4713	4674
Stk10	4674 / 4713	4674 / 4713	4674
Stk16	4713	4713	4674
Stk17b	4674 / 4713	4713	4674
Syk	4674 / 4713	4674 / 4713	4713
Tbk1	4674 / 4713	4674 / 4713	4674
Tec	4674	4674	4713
Tek	4674	4674	4713
Tgfbr1	4713	4713	4674
Tgfbr2	4713	4713	4674
Tie1	4674	4674	4713
Tlk1	4674 / 4713	4713	4674
Tlk2	4713	4713	4674
Tnk1	4674	4674	4713
Tnk2	4674 / 4713	4674 / 4713	4713
Tssk1	4713	4713	4674
Tssk2	4713	4713	4674
Yes1	4674	4713	4713
Zap70	4713	4713	4713

Legend for Supplementary Table 6:

The UniProt and AmiGO annotations for the Rat kinase proteins with Mouse orthologs

In this study 136 rat proteins had a mouse ortholog with a "potentially incorrect" AmiGO annotation. This table displays the Gene ID for the mouse and rat (they are the same because they are orthologs), the mouse GO label provided by AmiGO, the rat GO label by ISS (inferred from sequence similarity) with the mouse protein, and the corresponding UniProt annotation.

The ***Mouse/Rat Gene ID*** was obtained from each of the AmiGO protein records. The ***Mouse AmiGO Label*** field is "4713" (Tyr) if a query in AmiGO for the GO label GO0004713 returns the corresponding protein for mouse proteins, "4674" (Ser/Thr) if a query in AmiGO for the GO label GO0004674 returns the corresponding protein for mouse proteins, or "4674 / 4713" if a query in AmiGO for both GO labels GO0004674 and GO0004713 returns the corresponding protein. The ***Rat GO label by ISS Annotation*** field is "4713" if a query in AmiGO for the GO label GO0004713 returns the corresponding protein for rat proteins with an evidence code of ISS by association, "4674" if a query in AmiGO for the GO label GO0004674 returns the corresponding protein for rat proteins with an evidence code of ISS by association with a mouse protein or "4674 / 4713" if a query in AmiGO for the GO label GO0004674 and GO0004713 returns the corresponding protein for rat proteins with an evidence code of ISS by association with a mouse protein. The ***UniProt Label for Mouse*** field is " 4713" if a search in UniProt with the AmiGO Gene ID returns a mouse protein that contains a reference to the functional class "protein-tyrosine kinase activity", "4674" if a search in UniProt with the AmiGO Gene ID returns a mouse protein that contains a reference to the

functional class “serine/threonine kinase activity”, or “4674 / 4713” if a search in UniProt returns a mouse protein that contains a reference to the functional class “serine/threonine kinase activity” and “protein-tyrosine kinase activity” or any evidence that would suggest dual specificity.
