# Supplementary material for: Exploring inconsistencies in genome-wide protein function annotations: a machine learning approach
Source: BMC Bioinformatics. 2007 Aug 3;8:284. doi: 10.1186/1471-2105-8-284 (PMC1994202; doi:10.1186/1471-2105-8-284)
Supplement: Additional file 9 — Supplementary Table 7: Distribution of protein classes for human and mouse proteins annotated by AmiGO, UniProt, and HDTree. This table is a representation of the data used in Figure 1 which is a pie chart showing the distribution of human and mouse protein classes based on annotations found in AmiGO, UniProt, and predicted by HDTree. [file 1471-2105-8-284-S9.pdf]

Supplementary Table 7:

Species (Data Source)	0004674 Ser/Thr Kinase	0004713 Tyr Kinase	0004674 and 0004713 Dual Specific
Human (AmiGO)	233 (70.6%)	90 (27.3%)	7 (2.1%)
Human (UniProt)	233 (70.6%)	90 (27.3%)	7 (2.1%)
Human (HDTree)	230 (69.7%)	67 (20.3%)	33 (10.0%)
Mouse (AmiGO)	71 (29.1%)	106 (43.4%)	67 (27.5%)
Mouse (UniProt)	168 (68.9%)	65 (26.6%)	11 (4.5%)
Mouse (HDTree)	174 (71.3%)	66 (27.0%)	4 (1.6%)

Legend for Supplementary Table 7:

Distribution of protein classes for Human and Mouse proteins annotated by AmiGO, UniProt, and HDTree.

The table shows the distributions of each class of kinases (GO0004674, GO0004713, and Dual Specificity) for proteins retrieved from AmiGO, verified by UniProt, and predicted by the HDTree method. Each entry contains the number of proteins that belongs to the given class and its percentage compared to all the kinases for the given source. The Human dataset contains 330 proteins and the Mouse dataset contains 244 proteins. A pie chart of these distributions is shown in **Figure 1.**
